# Metabolic syndrome therapy in pediatric age — between classic and modern. From diets to pipeline drugs

**DOI:** 10.3389/fnut.2024.1475111

**Published:** 2024-12-11

**Authors:** Otilia Elena Frăsinariu, Vasile Valeriu Lupu, Laura Mihaela Trandafir, Violeta Streanga, Elena Jechel, Iris Bararu-Bojan, Ioana Vasiliu, Magdalena Cuciureanu, Isabela Ioana Loghin, Costica Mitrofan, Alin Horatiu Nedelcu, Anton Knieling, Ancuta Lupu

**Affiliations:** ^1^Faculty of Medicine, Pediatrics, “Grigore T. Popa” University of Medicine and Pharmacy, Iași, Romania; ^2^Faculty of Medicine, “Grigore T. Popa” University of Medicine and Pharmacy, Iași, Romania

**Keywords:** metabolic syndrome, child, pathophysiology, complementary therapies, lifestyle modification, Mediterranean diet

## Abstract

The metabolic syndrome, made up of the sum of the entities that define it (obesity, hypertension, dyslipidemias and non-alcoholic hepatic steatosis) has gained an important place in the research of the last decades. This aspect is mainly due to the complexity of management in pediatric practice. The main directions in his approach therefore bring together the concern of counteracting the noise or systemic, of the multiple intercurrents at the physiopathological level, as well as the negative imprint exerted on the quality of life. Its appearance and evolution are currently controversial topics, but the influence of genetic predisposition and lifestyle (diet, physical activity, psychological balance) are certainties. Considering the escalation of the incident at the global level, it is self-evident that it is necessary to know the pathogenesis and practice countermeasures for prophylactic or therapeutic purposes. The present work aims to summarize general aspects related to the metabolic syndrome encountered in pediatric age, with an emphasis on complementary therapeutic perspectives and their effectiveness, by analyzing the latest data from the specialized literature, accessed with the help of international databases (e.g., PubMed, Web of Science, Scopus, Embase, Google Scholar).

## 1 Introduction

The pediatric metabolic syndrome (MetS) is currently considered a pivot in the long-term development of diabetes and cardiovascular disease. Pathophysiologically, pediatric MetS represents an entity that has its main origin in the disruption of cellular metabolism and body weight, with different impact in pre- and post-puberty. Systemic consequences of pediatric MetS are characterized by the maintenance of insulin resistance and the promotion of systemic inflammation. Compared to the MetS approach in adulthood (e.g., risk and severity scores, therapeutic efficacy), the means of assessment in pediatrics are not yet completely standardized. Thus, in the current nutrition of the pediatric population, adaptations of the guidelines in force certified for adults are used, depending on pediatric values. Likewise, other variables included are reporting to the population-specific Z-score or correlation with markers of pathological processes (C-reactive protein, insulin, lipid profile, adiponectin, uric acid) (1, 2). For an easier diagnosis, in children, the pediatric MetS is defined by the presence of abdominal obesity (≥ 90th percentile), doubled by at least two of: high triglycerides (≥ 110 mg/dL), low HDL cholesterol (≤ 40 mg/dL), high blood pressure (≥ 90% for age, sex, height) or high blood sugar (≥100 mg/dL) (1). The estimated prevalence of pediatric MetS reaches a rate of up to 60% in the case of children with a very high body mass index (BMI). It is also noted that the impact of morbidity and mortality among these children has the potential to surpass even that of the parents (3).

The clinical importance of diagnosing pediatric MetS should not be underestimated. Considering the escalating incidence of obesity and sedentary behavior among young people, early recognition of MetS in children offers the opportunity for early interventions through lifestyle modification, thus preventing the development of associated chronic conditions (e.g., type 2 diabetes, cardiovascular diseases). Also, this aspect helps to reduce the therapeutic need, thus diminishing the negative effects of long-term use of pharmacological substances. Last but not least, the appropriate management of MetS in childhood contributes to the reduction of stigmatization, body dysphoria, and other psychosocial difficulties. Thus, we support the importance of monitoring metabolic risk factors in the pediatric population, when the ultimate goal is prompt detection and intervention to reduce associated morbidity and mortality (4, 5). Given the uncertainty of the diagnostic limits, the consensus was reached to identify and counter the risk factors grouped around weight gain and changes in insulin sensitivity, together with related comorbidities (e.g., non-alcoholic fatty liver disease, sleep apnea syndrome or that of polycystic ovaries, among teenage girls), rather than limiting to a single type of medication administered with a curative visa (6). In order to reduce the quality of life, multiple therapeutic options have been imagined, mainly centered on the removal of obesity, starting from the promotion of a healthy lifestyle, antidiabetic agents and culminating in bariatric surgery, the component that addresses a narrow range of patients, with criteria to include good stability, partly because of the peri-operative risks to which they are subject (7).

Useful to know in this sense, we consider the results obtained by He et al. with the help of a randomized study designed to evaluate the impact of a low-carbohydrate diet, a time-restricted food consumption, but also the combination of the two in the evolution of body weight, adiposity and biological constants in the metabolic sphere (fasting blood glucose, uric acid and lipid profile). The results reiterate the indisputable benefit of the combination of the two, while, viewed separately, the modification of the food constituents can only affect the BMI and the subcutaneous fat mass, with no effect on the visceral one or the biological profile (8).

Continuing the previously stated direction, we propose that through our manuscript we draw general lines in the understanding of pediatric MetS and its individualized, multidisciplinary approach. We therefore focus our attention on the risk factors involved in the initiation and maintenance of pediatric MetS, as well as on the clinical aspects and the current diagnostic implications of the main entities that make up the syn-drome. Also, an apparent place is assigned to therapeutic principles approved in the literature for pediatric MetS. In this chapter, we mainly focus on the impact of lifestyle changes, realized by adapting the diet, modulating the intestinal microbiota, as well as promoting a regular physical activity in reversing the metabolic decline and reducing the implicit systemic consequences. In order to achieve our goal, we proceeded to carry out a narrative review work. The development of research directions was achieved by querying the most important international databases (e.g., PubMed, Web of Science, Scopus, Embase, Google Scholar) regarding reference and current results in the field. For this purpose, we used key words and their combinations in the form of suggestive expressions for the chosen topic such as “metabolic syndrome,” “child,” “pathophysiology,” “treatment,” “lifestyle,” “diet modulation,” “diet Mediterranean,” “alternative therapies.”

## 2 Epidemiology of pediatric MetS

Having a variable prevalence, depending on the criteria used for diagnostic purposes, the age of the patients (between 1.2–9.8% among adolescents, compared to 0.2–1.2% in preschool and school-aged children), gender their (higher among boys) or geographic affiliation (the white and Hispanic population is more affected than African Americans, in contradiction to the individual prevalence of MetS components), this pathology is much more common among Americans and people from the Middle East, unlike Europeans and those from the Far East. The ratio is partly justified from the perspective of different food patterns between regions (1, 9). Geographical variability was brought to the fore by Noubiap et al. what, with the help of a study of specialized literature published until the beginning of 2021, carried out on patients aged 6–18 years diagnosed on the basis of blood pressure values (systolic and diastolic), waist circumference, plasma triglycerides and high-density lipoproteins high molecular, reiterated the reference ranges of MetS prevalence, extrapolating them according to regions, at the same time contradicting the direct link between the level of economic development and prevalence (10).

The most recent data from the literature, obtained by studying groups of patients belonging to low- and middle-income countries, report pediatric MetS at approximately 3% of the general population. However, among children with risk factors (e.g., overweight, obesity), the prevalence of the condition was reported in a percentage of 11–29% (11). Similar studies carried out in the United States of America report a correlation between lifestyle, unhealthy diets and the increased prevalence of MetS in adolescents. The authors attest to significant associations between high dietary inflammatory index and pediatric MetS components (e.g., increased waist circumference, decreased HDL cholesterol levels) (12). Also, the technological advance in the medical field led to the identification of some genetic factors (genetic polymorphisms) that can predispose children to MetS. Thus, fat mass and obesity-associated or cholesteryl ester transfer protein gene polymorphisms have been correlated with obesity and dyslipidemia, thus contributing to the escalation of pediatric MetS incidence. Therefore, the research of genetic factors represents a cornerstone in the prevention of MetS in the pediatric population (13).

The correlation between the comorbidities associated with pediatric MetS and the risk of its development is best exemplified in the case of obesity, where Guzmán-Guzmán et al. records a prevalence of 44.3% among children aged between 6 and 12 years and over-weight, strongly correlated with the manifestation of dyslipidemia, compared to only 0.84% among those who do not meet this criterion (14). In agreement with them, and Wan Mahmud Sabri et al. reports a prevalence of 56% of MetS in obese children, the factors strongly correlated with the development of the pathology being male sex, birth weight, lifestyle, hereditary maternal gestational diabetes, but also the impact of the subject’s age (the risk increases proportionally) or even the parental socio-economic level (15, 16). Unlike the change in body weight, the primary variations in blood pressure among adolescents are accompanied in 15–20% of cases by the development of pediatric MetS, the severity of its manifestation being directly correlated with metabolic disturbances. The two variables form a vicious circle, potentiating each other and causing changes in the target organs (17).

## 3 Risk factors and pathogenesis of pediatric MetS development

Although the main foundation in triggering the pathogenic cascade of pediatric MetS seems to be insulin resistance, the prevalence of MetS among subjects with impaired insulin sensitivity raises the suspicion of a combination of factors whose involvement conditions the development of the process. Among them we note the genetic component, doubled by the environmental characteristics (western diet, stress, sleep deprivation), ethnicity, sedentary lifestyle or tobacco consumption (18, 19). Wu et al. reports, through the analysis of cohort and cross-sectional studies published in the literature, a positive association between the level of physical activity practiced and the risk of MetS, variable correlation depending on gender (stronger among females), but also the link between it and the time spent in front of the screens (20).

With reference to birth weight, Bizerea-Moga et al. have objectified a correlation between this and the manifestation of obesity, hyperinsulinemia, impaired glucose tolerance and dyslipidemia, components strongly associated with pediatric MetS. In conclusion, they note that extremes of birth weight can be burdened later by an additional cardio-vascular risk. The prevalence of pediatric MetS was equally distributed between the two weight extremes (21). In determining it, an important role is played by the genetic component which, exercised in the form of transgenerational epigenetic inheritance, is influenced by maternal weight during pregnancy and breastfeeding (malnutrition or overweight), endocrinological disorders, hypoxia or exposure of maternal/paternal gametes to various environmental factors (e.g., smoking - modulates the risk of intrauterine growth restriction or congenital malformations, possibly through a toxic or hypoxic mechanism) that induce changes in the structure of DNA, RNA or histones (22). Low birth weight attributed to placental anomalies, the main maternal-fetal communication, seems to be associated with a recuperative weight gain during early childhood, an aspect that potentiates pediatric MetS comorbidities, but also with a reduced final height com-pared to population standards and pubertal anomalies or reproductive (early onset, testicular size and low testosterone levels, ovarian hyperandrogenism, risk of polycystic ovary) (23).

**Figure 1:**
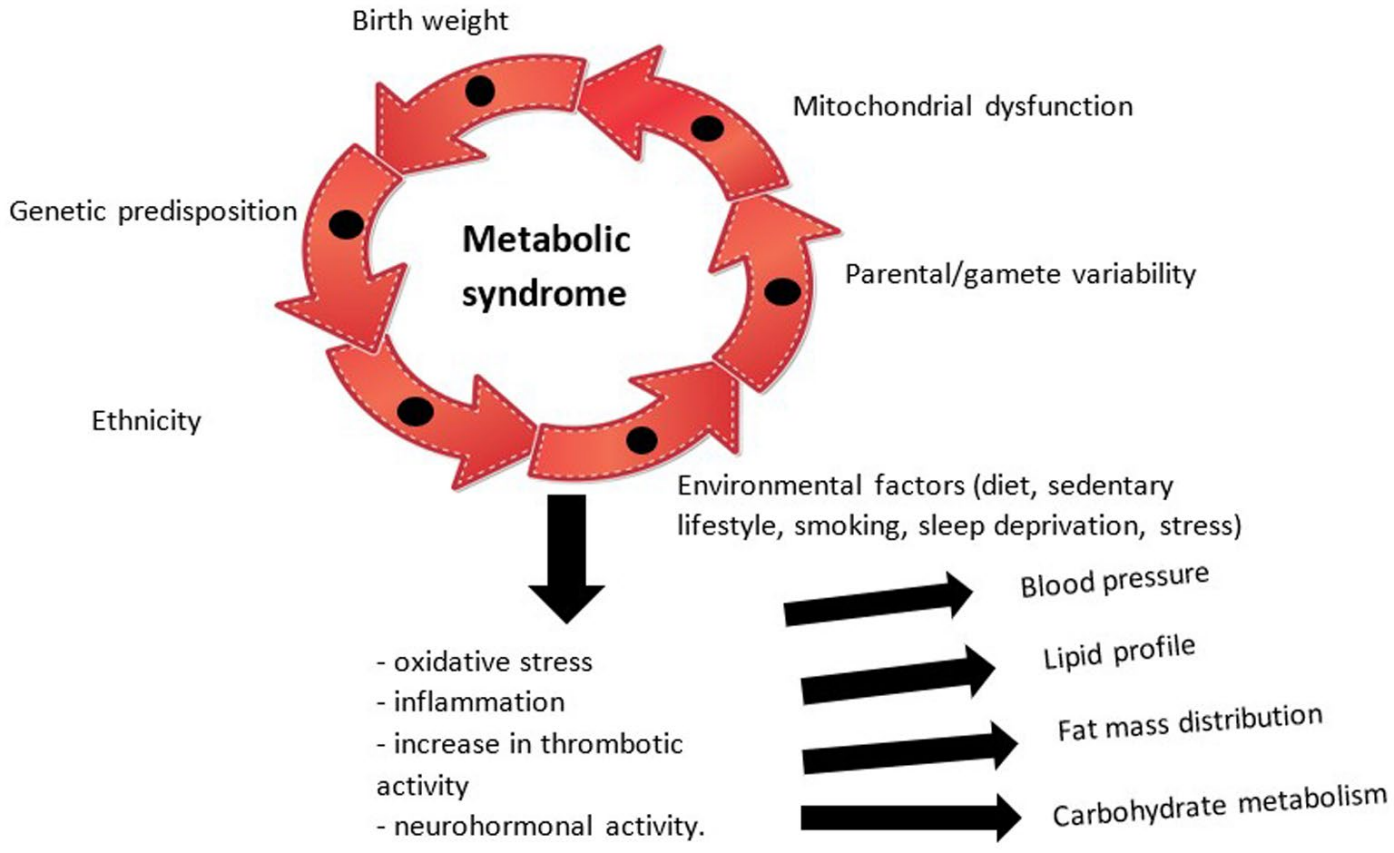
Pathogenesis of the metabolic syndrome.

The physiopathological mechanisms underlying the development of MetS at the pediatric age are therefore complex, briefly illustrated in Figure 1, whose exact chronological sequence is difficult to certify. Thus, through the accumulation of individual risk factors, modifiable or non-modifiable, a physiopathological cascade is initiated with neurohormonal effects (exerted by adipose tissue through leptin and adiponectin and the renin-angiotensin system), pro-inflammatory (the final pathway of all abnormalities, quantified with the help of tumor necrosis factor alpha -TNF alpha-, interleukin -IL-6 and C-reactive protein) and prothrombotic (correlated with the escalation of the fibrinogen value) which causes disturbances in the tensional balance, the distribution of fat mass, the lipid profile (with the appearance of atherosclerosis) and insulin sensitivity. All these entities currently do not benefit from a unitary therapy, but only from particular means of substitution and balancing (24, 25). Current research focuses on the influence of mitochondrial dysfunction in the occurrence of MetS, the hypothesis that opens new horizons in the approach to the condition and which is supported by the impact of oxidative stress and systemic inflammation in the pathogenic process (26).

## 4 Clinical aspects and diagnostic implications

The diagnosis of MetS in children remains a contradiction in the medical world, various hypotheses and limits being issued in its certification and implicitly in its constituent components. Currently, the consensus has been reached that obesity, as the first triggering factor of the process, is defined and framed according to the standard deviation of the BMI, related to age and gender. Additionally, the cardiometabolic damage is objectified by the presence of dyslipidemia (high molecular density lipoproteins - HDL < 40 mg/dL, triglycerides >150 mg/dL), fasting blood glucose >100 mg/dL and changes in insulinemia and systolic and diastolic blood pressure, with reported values that exceed the 90th percentile (27, 28). Considering the major impact of pediatric MetS on public health systems, it is necessary to develop effective screening and prophylaxis pro-grams among the population. Lopez Lucas et al. concludes, by analyzing a group of 3,325 children, that defining severe obesity as a deviation of +3 standard deviations from the BMI curve allows for the optimal identification of subjects with cardiometabolic disorders and thus the optimal concentration of resources (29).

From a clinical point of view, the brands of pediatric MetS are vast, but the most common and worth knowing by clinicians are represented by acanthosis nigricans (dermatomyositis frequently identified on the skin of obese people, with diabetes, but also in polycystic ovary syndrome, localized at the level of the armpit, the back of the neck or on the folds of the skin), stretch marks, signs of virilization, hepatomegaly, sleep apnea (intermittent obstruction of the upper respiratory tract that causes hypoxia and sleep disturbances), polyuria, polydipsia, polyphagia. There is also a close correlation between pediatric MetS and chronic inflammatory skin pathologies such as psoriasis, hidradenitis suppurativa and atopic dermatitis. It is also vital that at the time of the anamnesis the possible use of corticotherapy, antipsychotic drugs or thyroid dysfunction is excluded, conditions known to precipitate weight gain and thus influencing the objective clinical diagnosis (30–33). To facilitate the diagnosis of pediatric MetS from the perspective of its constitutive components, Table 1 summarizes the main lines currently approved by international guidelines.

**Table 1:** Pediatric MetS component diagnostic criteria.

Disease	Criteria	Reference
Obesity	WC or WHR BMI Z-score adjusted according to age and sex VAI: adapted according to gender and calculated based on anthropometric values and biological constants ABSI	Vizzuso S. et al. (34)
Dyslipidemia	Dosing of triglycerides, total cholesterol and its fractions (low or high molecular weight lipoproteins) Dosage of apoproteins Exclusion of cases of familial hypercholesterolemia	Guardamagna O. et al. (35) and Medeiros AM. et al. (36)
Hypertension	Optimal blood pressure measurement and classification based on nomograms Evaluation of target organ damage Exclusion of primary arterial hypertension (coarctation of the aorta, kidney disease, adrenal hyperplasia, pheochromocytoma, thyrotoxicosis, iatrogenic causes)	de Simone G. et al. (37)
Alteration of insulin metabolism and diabetes	Fasting blood glucose HOMA-IR HOMA-*β* QUICKI TyG ratio: evaluates muscle resistance to the action of glucose Genotyping in order to exclude insulin resistance syndromes of genetic origin	Vizzuso S. et al. (34) and Ogawa W. et al. (38)
Metabolic fatty liver disease	Dosage of liver enzymes (ALT and AST) GGT dosage Dosage of FGF21 Abdominal echography Elastography Liver biopsy puncture Investigations can be added for the purpose of the differential diagnosis of the causes of steatohepatitis: serology of hepatitis C virus (immunoglobulin G anti HCV), cytomegalovirus, Epstein–Barr virus, alpha-1 antitrypsin deficiency, thyroid hormone profile, screening tests for celiac disease (anti-transglutaminase IgA and IgG antibodies, anti-endomysium, deamidated anti-gliadin, duodenal endoscopy with biopsy), hemochromatosis (ferritin, transferrin saturation, genotyping), Wilson’s disease (ophthalmological examination, ceruloplasmin dosage, cupremia, copperuria), as well as toxicological dosages	Liebe R. et al. (39) and Brecelj J. et al. (40)

WC, waist circumference; WHR, waist-height ratio; BMI, body mass index; VAI, visceral adiposity index; ABSI, body shape index; HOMA-IR, homeostatic model for insulin resistance; HOMA-β, HOMA index for beta cell function; QUICKI, quantitative insulin sensitivity verification index; TyG, triglyceride-glucose; ALT, alanine aminotransferase; AST, aspartate aminotransferase; GGT, gammaglutamyltransferase; FGF21, fibroblast growth factor 21.

## 5 Therapeutic perspectives

The management of pediatric MetS occupies the most important role in the evolutionary course of patients diagnosed with this condition. Both from the perspective of the psychologically delicate period that the subjects go through, starting with late childhood and culminating with adolescence, as well as due to the impact played by a harmonious development in defining the later adult, current studies promote the approach on several levels, in order obtaining the best results. Worth mentioning is the observed correlation between the child’s temperament at various ages and the risk of developing pediatric MetS - the link observed in the case of hyperreactivity and negative behavior, marked by an escalation of aggression or anger, mainly in males. Attention is thus drawn to the role played by the psychologist and the pediatric psychiatrist within the multidisciplinary team (41).

Being a pathology developed mainly due to the predisposition toward obesity and multisystemic damage encountered predominantly in the first part of life, it is self-evident that the preference toward an initial prophylactic/"non-invasive” approach, in the hope of regulating the clinical-biological parameters with the help of promoting a healthy lifestyle (diet, physical exercises, psychological support). In case of failure of these measures, the recommendations advance by introducing medicinal substances depending on the objective comorbidities (1, 42). The non-pharmacological and pharmacological principles used in the adjunctive management of pediatric MetS are briefly developed in Table 2. Next, summarizing the systemic consequences of pediatric MetS, Urbina E. emphasizes the imprint of left ventricular dimensions and diastolic function, changes in intimal thickness, endothelial function and arterial stiffness, microalbuminuria, steatohepatitis, polycystic ovary syndrome, orthopedic conditions, sleep apnea, eye damage or of the peripheral nervous system (43).

**Table 2:** Therapeutic principles in the metabolic syndrome.

Therapeutic lines	Principles used
Dietary regime	Caloric Restrictions decreasing the intake of sugars, fats, sodium diet based on fruits, vegetables, olive oil, fiber diet rich in omega 9 fatty acids, proteins, zinc, selenium, flavonoids, antioxidants (vitamin E, C, beta-carotene), vitamin B12, folate, magnesium
Physical activity	It is recommended to practice moderate physical activity, according to tolerance, for 30–60 min a day, after the age of 5 years
Integrity of the intestinal microbiota	Balanced diet, rich in fiber prebiotics, probiotics, symbiotics transplantation of fecal matter
Excess weight	Diet, physical activity (previously described) Pharmacological treatment (Orlestin, Sibutramine**, catecholaminergic agents**, Metformin, Octreotide**, Topiramate**, Fluoxetine**) Bariatric surgery
Alteration of glucose and insulin metabolism	Adequate rest, limiting exposure to screens Metformin Sulfonylureas **Insulin Sodium-glucose cotransporter 2 (SGLT2) and dipeptyl peptidase 4 (DPP4) inhibitors*
Disturbance of the lipid profile	Cholestyramine Colestipol Ezetimibe Statins (Atorvastatin, Rosuvastatin) Fibrate, Nicotinic acid **Colesevelam
Tension balance	Angiotensin-converting enzyme inhibitors Angiotensin receptor blockers Calcium channel blockers Thiazide diuretics Endothelin receptor antagonists (Darusentan) *Renin inhibitors (Aliskiren)*
Combating non-alcoholic hepatic steatosis	Approaching a healthy lifestyle Combating obesity Probiotics, N-acetyl cysteine, anti-diabetics, omega 3 fatty acids or bile acids, vitamins E and D (in research)

SGLT2, sodium-glucose cotransporter 2, DPP4: dipeptyl peptidase 4 inhibitors. *Drugs considered off-label, under research; **Medicines used with caution, only in special situations.

### 5.1 Diet

As the parallel existing between MetS and pediatric obesity is well known, the diet indicated among this group of patients must be a balanced one, based on the exclusion of products rich in saturated fats, processed foods, sodium, sugars (which induce an increase in insulin production in the pancreas), carbohydrates or with a high caloric content. A Mediterranean diet (MD) is thus preferred. Additionally, we note the importance of encouraging the practice of physical activity. We recognize the difficulty of obtaining and maintaining these goals both in the pediatric population and in adults, partly due to low motivation (1, 44–46). Therefore, in order to achieve a healthy development of children and to prevent long-term metabolic and cardiovascular diseases, we consider it important to know the basic principles of the Mediterranean diet. Being one of the most intensively studied diets, the Mediterranean food model is centered on high fiber consumption in the form of fruits and vegetables (essential source of vitamins, minerals and antioxidants), the preference for using olive oil as the main source of monounsaturated fats and antioxidants and grain integration, moderate consumption of proteins, especially fish (source of omega-3 fatty acids) and white meat, increased consumption of nuts and legumes (e.g., beans, lentils, chickpeas), limited consumption of sweets and sweetened beverages, adequate hydration with water, regular meals with moderate portions and regular physical activity (47, 48). Currently, the optimal integration of MD into the lifestyle must also take into account the entire process of food production and processing (e.g., the harvesting process), the characteristics of food consumption (e.g., seasonal and local consumption), cooking techniques (extensive use of olive oil with different spices) or eating behaviors (social consumption) (49).

Adherence to such a diet has multiple benefits, among which we note the reduction of obesity, carbohydrate/lipid metabolic disorders and the prevalence of comorbidities associated with pediatric MetS - an aspect certified by the randomized controlled study of 70 teenage girls (50). Also, the current literature notes the relationship between low MD adherence and increased incidence of central obesity, hypertriglyceridemia, and insulin resistance. However, data on the implications of obesity-related genotypes in modulating the relationship MD adherence – obesity – pediatric MetS risk are considered insufficient. Thus, the authors encourage the continuation of research in this field (49).

Another vital aspect of the preference for a MD is attributed to the reduction of chronic systemic inflammation by increasing the amount of antioxidants and healthy fats ingested. Through this mechanism, MD intervenes in regulating lipid homeostasis and reducing the risk of cardiovascular diseases (51, 52). At the same time, MD was correlated with an improvement in insulin sensitivity and a decrease in blood glucose levels, an essential aspect in the prevention and treatment of type 2 diabetes in children and adolescents (47, 53).

In order to be able to adapt and monitor adherence to the Mediterranean dietary changes according to individual needs, the authors have imagined over time several evaluation models. Among these, we mention the KIDMED Mediterranean diet quality index for children and adolescents as a benchmark (54). Cars et al. recognize the indisputable benefits of MD, doubled by avoiding a sedentary lifestyle in increasing the quality of life of children. Next, the authors discover incompletely studied directions. In this sense, they support the need to direct future efforts toward exploring the impact of MD on cognitive functions, academic performance and mental health, emphasizing the importance of establishing healthy eating habits early in life (51).

Among all food constituents, the most important contribution in pediatric MetS, insulin resistance and waist-to-hip ratio was played by solid fibers that interact with adipose balance by slowing gastric emptying and promoting satiety, Ventura et al. emphasizing that, 1.1 g of fiber (one apple) represents the difference in value between the diet of children without symptoms of MetS and that of subjects manifesting this condition (55).

**Figure 2:**
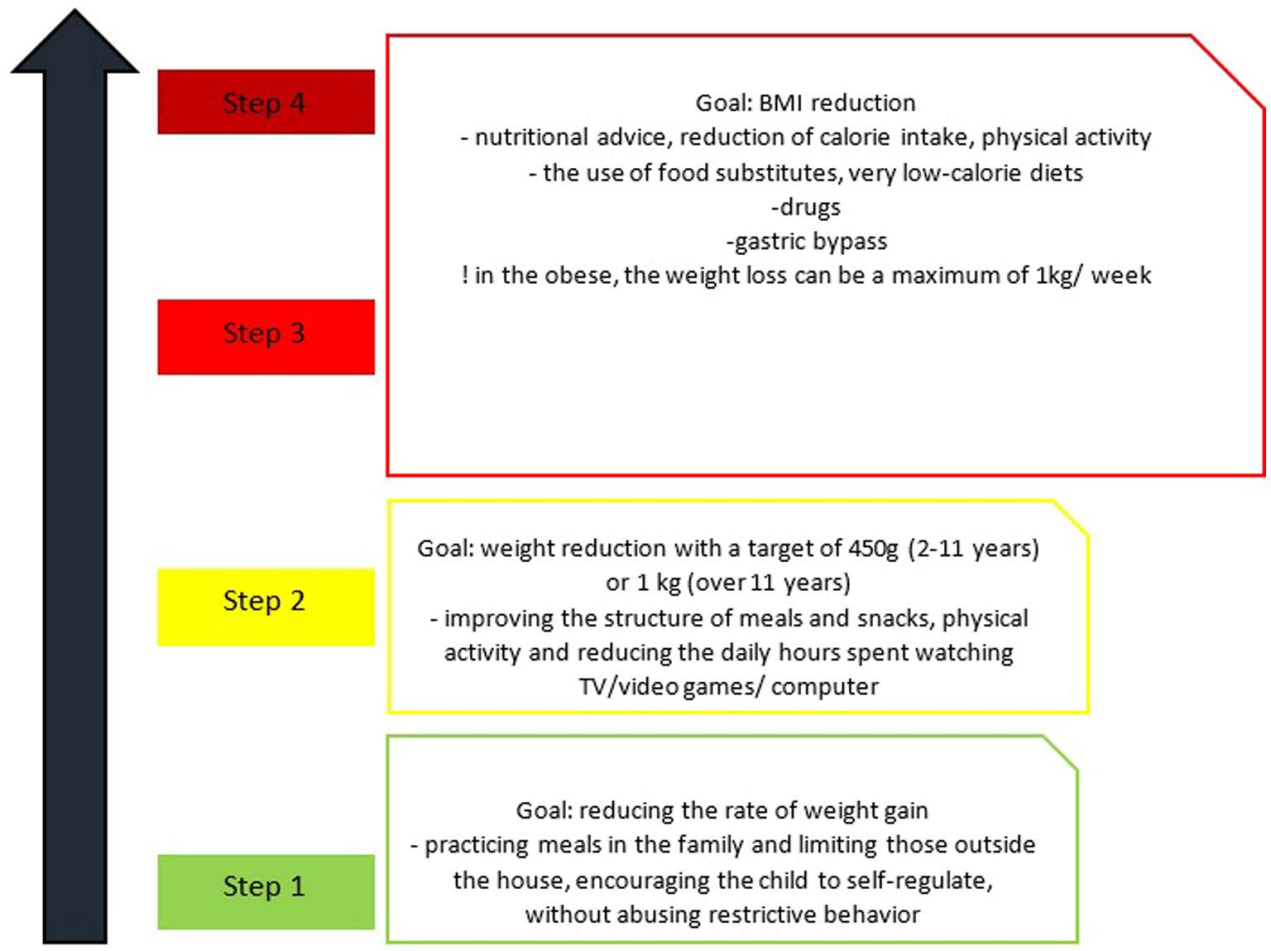
Stages of dietary change in metabolic syndrome in children (56).

Thus, the weight loss obtained with the help of non-surgical means has been proven to have a beneficial effect on systemic inflammation, arterial hypertension, dyslipidemia and insulin resistance, although it is difficult to know exactly the percentage of weight loss required for each child depending on age, gender and stage of puberty (44, 56). For the purpose of easier orientation, the dietary treatment of pediatric MetS has been subdivided into four steps (Figure 2).

It seems that the relationship between pediatric MetS and obesity - a condition whose incidence is predominantly dictated by eating habits and daily physical activity - is a bidirectional one. The latter exerts both systemic effects, potentiating the incidence of pediatric MetS and the main comorbidities that make it up, as well as individual effects on each individual component (57, 58). In support of the correlation stated above, we mention the results of a randomized study carried out by Velázquez-López et al., which objectified the different impact of the two types of diet (Mediterranean versus fast-food type) on BMI, blood glucose level, triglycerides and pediatric MetS components. The authors note an improvement in these variables after 16 weeks of a diet rich in dietary fiber, omega 9 fatty acids, proteins, zinc, selenium, vitamin E and flavonoids. Additionally, an increase in HDL-cholesterol is observed (59). Also, Pedrosa et al. identifies a decrease in the prevalence of MetS by 1.6% among overweight/obese children aged between 7 and 9 who followed a physical and nutritional counseling program during 1 year. In the same category, the authors reiterate the improvement of the previously specified parameters (60).

Diet also modulates inflammatory activity. Dietary intake of fats and antioxidants (vitamin E, C and beta-carotene) was noted as an important factor in the dynamics of C-reactive protein (CRP), interleukin 6 (IL-6), leptin and retinol-binding protein levels (RBP) 4. No similar findings were issued in the case of tumor necrosis factor (TNF-alpha) (50, 61). Besides these, the level of vitamin B12 correlates inversely proportionally with the risk of MetS in children, while the folate value is directly proportionally correlated. Vitamin B6 does not seem to influence this pathology (62). Another important micronutrient in the pathogenesis of pediatric MetS, type 2 diabetes and obesity is magnesium. However, its deficit is underestimated, partly due to the uneven distribution between the plasma and the intracellular environment, an aspect that can vitiate its dosage. We also draw attention to the calcium–magnesium interrelationship, crucial both in obesity and pediatric MetS, as well as in insulin homeostasis (correlated inversely proportionally with the value of magnesium) (63).

Despite the multiple advantages stated previously (e.g., improving insulin sensitivity, reducing body weight, increasing the intake of antioxidants and fibers, improving the lipid profile or the blood pressure level) it must be recognized that the diets frequently used in the management of MetS in children can be burdened by various limitations (64, 65). Among these we note aspects such as the risk of deficiencies in fiber, vitamins from the B complex and antioxidants, found in the case of diets low in carbohydrates. This can also predispose to an unbalanced diet that will subsequently increase the risk of dyslipidemia or cardiovascular diseases. In parallel with this, diets based on fat reduction can potentiate deficiencies of fat-soluble vitamins (A, D, E, and K) and essential fatty acids. These biomolecules are essential for the optimal neurological and immune development of children. At the same time, low fat intake can predispose to an increased consumption of refined carbohydrates, which aggravates imbalances in carbohydrate metabolism (12, 66–68). Finally, regarding the Mediterranean diet, we would like to draw attention to the fact that, although it is considered well tolerated, accompanied by low risks, it can be difficult to maintain due to cultural considerations or various restrictive culinary preferences of children. Also, monitoring caloric intake is important because excessive consumption of fats, even healthy ones, can stimulate weight gain. Similarly, the DASH diet can be assimilated as a diet that is difficult to follow and calibrated so as not to reduce the intake of healthy fats and proteins below the optimal level, essential elements for the normal growth and development of children (69, 70).

### 5.2 Physical activity

Progressive, moderate, non-competitive physical activity (e.g., using a bicycle for transportation, walking, jogging, and swimming, joining sports clubs) is inversely correlated with the risk of developing pediatric MetS. It is thus recommended a minimum of 30–60 min/day for children older than 5 years, with proven importance in increasing basal metabolism and maintaining the balance between ingested energy and consumed energy, reducing BMI and waist circumference (more pronounced in the case of boys), of the substrate necessary for lipogenesis, insulin resistance and systemic inflammation, the inversion of the HDL/LDL and triglyceride ratio and the improvement of endothelial function in parallel with the improvement of blood pressure. However, less than 30% (between 20–80%) of patients manage to reach this target (1, 44, 56). It is important to mention that exercises that cause increased or repeated pressure on the joints of the legs or hips should be avoided in the case of children with severe obesity (44).

With regard to the triad of fats — pediatric MetS — physically activated, it seems that the first ones show a tighter correlation, objectifying the continuation of mutual modulation despite the practice of physical activity. This particularity was not confirmed in the fitness - pediatric MetS correlation. In this situation, the relationship becomes insignificant after controlling for obesity (71). In this sense, we rather emphasize the importance of practicing physical activity for preventive purposes, for modulating the oxidation of the carbohydrate substrate (important in the Apoprotein B-Apoprotein A1 balance) and lipids, maintaining weight in optimal ranges and regulating insulin sensitivity through the influence exerted on proteins and receptors responsible for glucose transport (GLUT4). Although it is unanimously recognized that the two components - diet and sport - act partially synergistically, the difference between them consists in the impact brought by physical activity on muscle mass (72–75). Regarding the type of physical activity practiced, it is difficult to delimit the benefit brought by aerobic versus resistance exercises, the two addressing similar mechanisms such as the promotion of angiogenesis at the muscle level, the increase of blood flow, the stimulation of GLUT and adrenergic receptors (alpha and beta) and the regulation of carbohydrate and lipid metabolism. Their combination is therefore an optimal option (74, 76). However, the ratio between the two types of physical activities is not unanimously recognized. His reports vary in the literature between the practice of both equally and the predominance of aerobic activity. Currently, it is postulated that the preference for a certain aerobic/resistance ratio must vary depending on the severity of the symptoms, the targeted objectives (weight loss and improvement of cardiovascular capacity versus increase of muscle mass and improvement of metabolic rate) and the physical condition of each child (76–78). Another effect of resistance physical activity is represented by the influence of the intestinal microbiome, the structure in a fine balance that imprints both the internal homeostasis, as well as the therapeutic course of numerous pathologies, among which we define pediatric MetS (79).

### 5.3 Balance of intestinal microbiota

The intestinal microbiome represents a biosystem made up of trillions of micro-organisms (bacteria, viruses and fungi) that is in a perpetual evolution regarding the diversity and abundance of bacterial genera from the intrauterine environment until the age of senescence (80). Together with the vital organs (e.g., heart, lung, kidney, skin) it forms numerous links whose disturbances impact the homeostasis of the internal environment at various levels (e.g., inflammatory, atopic, autoimmune) (81–89). The current medical literature notes that the alteration of this microenvironment produced by a diet rich in fats, in favor of fibers, is therefore called “intestinal dysbiosis.” Under the umbrella of this definition, we find the abundance of harmful bacterial species (*Enterobacteriaceae, Staphylococcus* and *Clostridium perfringens*) at the expense of beneficial ones (*Lactobacillus, Bifidobacterium*), a balance that influences numerous pathologies both in the sphere of the nervous system and the digestive system, as well as among those that make up pediatric MetS (e.g., obesity, hypertension, type 2 diabetes) (90–93). It is also reiterated the increased risk of colonization with pro-inflammatory or entero-toxic *Escherichia Coli* in the case of diets rich in fats and sugars (93).

In the pathophysiology of intestinal dysbiosis, a key role is played by short-chain fatty acids (acetate, propionate, butyrate) resulting from fermentation processes and which, in addition to being a powerful metabolic and defense source for epithelial cells, regulate the production of YY peptides and GLP-1 with a role in modulating food intake and the feeling of hunger, but also insulin production and sensitization to it. Added to these are the influence of molecular models associated with pathogens, bile acids, branched chain amino acids and colonic gasses (90).

In the light of these findings, the involvement of the modulation of the human microbiome in the prevention and treatment of pediatric MetS-related disorders is self-evident. This can be achieved with the help of prebiotics from food sources such as asparagus, garlic, leeks, onions, bananas, chicory, wheat bran, and barley. Prebiotics have a role in favoring the preferential growth of commensal bacterial strains. Other means used are represented by probiotics (live microorganisms -e.g., bacteria-, administered exogenously, which contribute to the balance of the intestinal microbiota and the optimal functioning of the digestive and immune systems) or symbiotics (mixtures of prebiotics and probiotics). Although they show heterogeneous results depending on the strain used, the dose, the treatment period and the individual characteristics of the patient at the beginning of the treatment, have proven effective in restoring the intestinal barrier, regulating metabolic parameters, body fat, serum glucose, inflammation and biological (transaminases) and histological characteristics of liver damage (83, 94–97). Although studies among pediatric patients are not as popular and easy as those that include adults, the data in the literature still note the benefit of *Lactobacillus* administration in the affected population/with risk factors, with the reversal of the ratio of harmful bacteria/beneficial bacteria and the increase in the level of acids short chain fats (90). A similar effect was also proven in the study of supplementation with *Bifidobacterium*, in murine models. In this situation, the reduction of intestinal inflammation and the improvement of metabolic imbalances were observed (98, 99). The therapeutic hypothesis was also certified by Pan et al., with the help of a meta-analysis. The authors add to the above the harmful impact of the acidic environment created in the intestine due to the administration of prebiotics on the species of *Clostridium perfringens* and *Escherichia Coli* (90).

Much more modern, but in the research stage in adults, fecal matter transplantation represents another short-term therapeutic option aimed at the intestinal microbiota. The current literature recognizes favorable results 6 weeks after implementation in terms of peripheral sensitivity to insulin. However, no effects were observed on fasting blood glucose, hepatic gluconeogenesis, BMI, or lipid profile. At the same time, an increase in *Ruminococcus bromii* and *Roseburia intestinalism*, bacterial species involved in the degradation of fibers and the production of short-chain fatty acids, was observed (100). In pediatric practice, it was introduced in 2010, being used in *Clostridium difficile* infection with minor adverse effects - bloating, diarrhea, constipation, abdominal pain, old age or transient fever. Major complications rarely occur, among which we mention aspiration pneumonia in the case of delivery techniques through the upper gastrointestinal floor. Other diseases in which modulation of the intestinal microbiota was practiced for therapeutic purposes were inflammatory bowel diseases, irritable bowel syndrome, refractory diarrhea, neurological diseases (Parkinson’s disease) and autism spectrum disorders (101).

### 5.4 Fighting obesity

The most important parameters influencing the development and course of MetS in the pediatric population, according to Hirschler et al., seem to be obesity and insulin resistance. The observation was reinforced by the results of a cross-sectional study on 1,009 children, aged between 6 and 14, attending elementary schools in Buenos Aires, Argentina, between April and September 2007 (102). The same author goes further in the study of the impact played by body mass in dictating the subsequent predisposition to comorbidities, reiterating the relationship between increased birth weight and the development of MetS and overweight/obesity in childhood. In contrast, low birth weight has no association with these (103). Obesity is assessed using the BMI Z-score, its increase by one point doubling the prevalence of pediatric MetS. Thus, Sen et al. emphasizes the importance of screening for MetS among children with a BMI Z-score > 2, with the aim of increasing early diagnosis and treatment (104). Also, an important role in optimal management is attributed to dietary modulation and counteracting the pro-oxidative status specific to the pathology (105).

The pharmacological therapy used in obesity is reduced in pediatric practice, partly due to the incomplete research of the products, currently only Orlestin is recommended - a drug that inhibits intestinal lipase, thus reducing the level of triglycerides, cholesterol and body weight by approximately 3%/month. It is approved among teenagers over 12 years old, presenting limited adverse effects such as steatorrhea, abdominal pain or a feeling of urgency to defecate. It is also burdened by the risk of affecting absorption through the interaction with fat-soluble vitamins. Other substances used are Sibutramine and catecholaminergic agents – diethylpropiona, fenproporex and mazindol (44, 45, 56, 106). The clinician’s choice of one of the previously mentioned drugs is dictated by the patient’s associated symptomatology. Thus, Orlestin is used in the case of high-fat diets, not before excluding severe gastrointestinal disorders. Sibutramine, in a dose of 10–15 mg, is indicated in the case of facing the lack of satiety, over the age of 16. Finally, catecholaminergic substances are preferred when pediatric MetS appears in association with attention deficit. There are also other drugs with anti-obesity effects but with restrictions regarding administration due to side effects and contraindications in certain pathologies. Among these we mention Topiramate (anticonvulsant, a history of kidney stones must be excluded), Bupropion (contraindicated in epilepsy) and Fluoxetine (selective serotonin reuptake inhibitor, not administered in bipolar disorders/epilepsy) (56, 106). It is recommended to stop the anti-obesity treatment if it did not have the effect of >4% decrease in BMI or Z-score after 12 weeks of administration at the maximum dose (45).

Metformin, the orally administered representative of the Biguanide class, is a hepatic modulator for insulin, inhibiting gluconeogenesis and thus positively impacting BMI, cholesterolemia and LDL-cholesterol level. At the same time, it is noted that it exerts cardioprotective effects. Thus, it represents a therapeutic option proven to be effective among adolescents, at a dose of 2 g/day (45, 56, 107). The indirect effects of Metformin are obtained by stimulating glucagon-like peptide 1 (GLP-1) agonists. Among the side effects we mention the digestive damage objectified by nausea, bloating, diarrhea or flatulence, vitamin B12 deficiency, lactic acidosis (encountered with the ad-ministration of the substance among patients with renal failure), cardiac damage (congestive heart failure, peripheral perfusion damage), pulmonary (hypoxia) and hepatic (106).

Alternative therapeutic lines seem to include Octreotide and Topiramate (0.5–22 mg). The first one targets pancreatic insulin secretion producing its suppression, with the consequent decrease in fat mass (up to 43% when associated with life-style changes) and the reduction of acanthosis nigricans. However, it is burdened by side effects such as gallstones, hypothyroidism, diabetes and impairment of cardiac function. Consequently, it is important to specify that the two substances are recommended to be used with caution, only in special situations and under careful monitoring (106).

Bariatric surgery is another therapeutic method used among adolescents who meet the criteria. Currently, these include a BMI over 35 kg/m2 accompanied by severe comorbidities such as type 2 diabetes, moderate/severe sleep apnea, liver fibrosis, brain tumors or disabling orthopedic conditions or over 40 kg/m2 together with mild comorbidities such as dyslipidemia, arterial hypertension, mild sleep apnea, steatohepatitis (44). Regarding the surgical techniques, the most often used is the vertical gastrectomy due to the low postoperative complications and the efficiency in weight loss. More recently, modern techniques such as the intragastric balloon or percutaneous gastrostomy are coming to the attention of researchers and medical practitioners, although the data on the benefit and safety of the practice in the pediatric population are insufficient (45). Bariatric surgery can induce metabolic disorders such as malnutrition, loss of bone mass, hyposideremia, hypocalcemia or vitamin deficiencies (A, D, E, K, B12) (106).

### 5.5 Correction of disorders in glucose and insulin metabolism

The therapy of insulin resistance (IR/metabolic syndrome X) encountered in pediatric MetS remains open to study. The condition is mainly defined by fasting hyperinsulinemia, decreased glucose tolerance, hypertension and dyslipidemia. Additionally, microalbuminuria, the inflammatory syndrome, but also the disruption of essential fatty acid metabolism can be associated (108, 109). Of all these, the most challenging is maintaining glycemic values between optimal levels. This is mainly due to multiple interactions with exogenous and endogenous factors (e.g., the recent COVID-19 pan-demic) (110). The administration of Metformin (in an initial dose of ½ of the adult dose, later increasing to 2/3 of the dose) or other pharmacological products is controversial, as IR has proven to be mostly transient. Less than 2% of subjects develop type 2 diabetes within 5 years. However, when it is installed, in addition to initiating a healthy lifestyle, the pharmacological component is represented by Metformin, Sulfonylurea and Insulin - alone or in combination, depending on the severity of the hyperglycemia, the HbA1c level and the presence/absence of ketosis and ketoacidosis. It should also be taken into account the need to monitor glycemic values and adapt therapeutic doses after correcting the basal glycemia (ideally with the help of the 8-point glycemic profile) (44, 56, 111).

The period of rest (sleep) has proven to be important both in the optimal growth and development of children, as well as in the modulation of insulin sensitivity. A rest period of 8 to 11 h is thus recommended, limiting exposure to screens (blue light) to less than 2 h/day (44). If we still consider the use of pharmacological therapy, it is vital to know certain principles regarding antidiabetics. Among these, we mention the fact that Metformin decreases the hepatic production of glucose, increases peripheral uptake and improves insulin resistance, still requiring caution in the case of teenagers due to its ability to induce ovulation, which can lead to an unwanted pregnancy. Sulfonylurea, administered in a dose of 2/3 of the dose recommended for adults, stimulates the increase of basal and postprandial insulin, with the risk of hypoglycemia. Last but not least, Insulin used in the form of medium- or long-acting analogs corrects blood sugar, reduces glucosuria and can induce hypoglycemia or weight gain (56). Other therapeutic options used in adults, but without sufficient data in children, are sodium-glucose transporter 2 (SGLT2) and dipeptyl-peptidase 4 (DPP4) inhibitors (45).

### 5.6 Regulation of lipid homeostasis

Lipid balance disturbances are defined as a decrease in HDL, with an increase in triglycerides and maintaining LDL in relatively normal values. Similar to the majority of components that make up pediatric MetS, in their treatment a central role is played by changing the diet with the aim of reducing cardiovascular risks. Note the decrease in LDL and total cholesterol obtained by the combination of reducing dietary fat to 25–30% of daily caloric intake, saturated fat to less than 10% and cholesterol intake to less than 300 mg/day. Diet modulation is preferred over pharmacological therapy with statins, bile salt chelators and cholesterol absorption inhibitors, considered reserved for severe cases or non-responsive to dietary measures (44, 112, 113). Other non-pharmacological therapeutic means are represented by capsules based on pasta oil, administered in a dose of 2 g/day (112).

The addition of the pharmacological component in the therapy of dyslipidemias is recommended to be done after the age of 8 years, in cases with LDL cholesterol ≥190 mg/dL or ≥ 160 mg/dL doubled by family risk factors such as early cardiovascular dis-eases or ≥ 130 mg/dL in the presence of diabetes. Pharmacological treatment under the age of 8 is reserved for cases with LDL cholesterol ≥500 mg/dL (45, 106). Worthy of emphasis is the escalation of the triglyceride value above the threshold of 500–700 mg/dL, at which level the institution of treatment is imperatively necessary due to the increased risk of pancreatitis (112, 114).

From a medicinal point of view, the first therapeutic lines are represented by cholestyramine (resin lacking intestinal absorption, with side effects on the absorption of fat-soluble vitamins and intestinal transit), colestipol, Ezetimibe (cholesterol absorption blocker by acting on the Niemann-Pick C1 protein -like 1) and statins. The side effects noted were similar to adult administration, without disturbing growth and cognitive and sexual development. Fibrate and nicotinic acid have conflicting recommendations regarding administration to children (44, 56, 106, 114). The most potent statins in pediatric treatment proved to be Rosuvastatin, followed by Atorvastatin, due to the prolonged half-life. About the two, it should be known that they can present numerous drug interactions (due to the use of the CYP3A4 substrate), but also side effects such as headaches, dizziness, myalgias and gastrointestinal symptoms (44). Statins produce a reduction of LDL by 30–40% and the risk of atherosclerosis (by reducing the intimal thickness at the level of the carotid vessels), consequently having an impact on the morbidity and mortality induced by cardiovascular diseases. The therapy should be initiated mainly in children older than 8 years to avoid the risk of affecting growth and sexual maturation. The dose used must be small, titrated according to the LDL value and carefully monitored in terms of hepato-renal function (stopping is recommended when liver enzymes exceed 3 times the normal value or creatine kinase exceeds 5 times the normal value) and muscle symptoms - skeletal (myalgia, muscle weakness) (106, 114, 115).

The modern therapies, newly introduced in the therapeutic schemes of dyslipidemias, target cholesterol ester transfer protein inhibitors (Torcetrapib) and Colesevelam (106).

### 5.7 Maintenance of tension levels

At the pediatric age, blood pressure assessment is done with the help of reporting the child’s value to specific percentiles for age, gender and height. Thus, a normal blood pressure value is defined as the systolic and diastolic pressure value below the 90th percentile for age, gender and height. Furthermore, current guidelines define “prehypertension” as systolic and diastolic blood pressure values greater than the 90th percentile, but less than the 95th percentile for age, gender, and height. Finally, hypertension in pediatrics is considered when the systolic and diastolic values exceed the 95th percentile for age, gender and height (116, 117). Blood pressure imprints the risk of developing pediatric MetS and cardiovascular mortality, along with obesity, increased insulin resistance, hyperinsulinemia, dyslipidemia, chronic inflammation or pro-oxidative status. Thus, a bidirectional relationship between the two entities takes shape. This is influenced, during childhood, by aspects such as practicing physical activity, a diet rich in fruits, vegetables, olive oil (source of polyphenols) and low in sodium. These variables can also be addressed later as the first line of therapy, preceding the introduction of pharmacological substances such as angiotensin-converting enzyme inhibitors - ACEI, thiazide diuretics, calcium channel blockers - CCB or angiotensin receptor blockers - ARB or in combination with them in order to obtain a maximum therapeutic effect (44, 45, 116, 118). In addition to the secondary etiology of hypertension in children (e.g., renal, endocrinological, cardiovascular, pharmacological, obstructive sleep apnea or neoplasia) are also its primary causes (e.g., environmental factors, family history, lifestyle). Also, genetic or prenatal factors (e.g., low birth weight, intrauterine growth restrictions and exposure to maternal smoking during pregnancy) are variables that can negatively impact the prevalence of hypertension in children. Finally, we do not omit the transient causes of hypertension such as fever, acute pain, stress or certain infections (119, 120). Correlations were also observed between the use of antihypertensive treatment in adolescents and regression in the size of the left ventricle, but without improvement in heart rate - rather correlated with visceral obesity that modulates the activity of the sympathetic nervous system (121).

Thus, cardiovascular prevention is an important mechanism in reducing associated morbidity and mortality. This can be done on several levels, the most important of which is primary prevention (preventing the appearance of risk factors for cardiovascular diseases before they develop) and primary (preventing the appearance of cardiovascular diseases in people who already have risk factors). If the first focuses on educating young people about the importance of a balanced diet, regular physical activity, avoiding smoking and limiting alcohol consumption, primary prevention involves monitoring and managing known risk factors through lifestyle changes and the use of pharmacological therapy to reduces cardiovascular risk (119, 122).

In the pre-hypertension phase, it is recommended to practice a healthy life-style, with the promotion of weight loss. For this purpose, a high blood pressure control diet (DASH) is imagined that follows the principles stated above — high intake of whole grains, vegetables, fruits, nuts, legumes, doubled by a moderate intake of low-fat and low-fat dairy products of red meat, sugary drinks and sodium. In the later phases, it is recommended to introduce individualized drug therapy depending on the blood pressure values (123, 124). Weight loss, objectified with the help of an approximately 10% lower BMI, leads to a reduction in blood pressure between 8 and 12 mmHg. Similar results were recorded in the case of practicing regular physical activity for 3–6 months (118). The pharmacological products used to modulate blood pressure must be carefully chosen so as not to interfere with insulin resistance, weight gain (e.g., thiazide diuretics) or glucose tolerance (e.g., beta-blockers). It is therefore understandable the importance of knowing the mechanisms of action, side effects and possible contraindications (56). Initial monotherapy is recommended, with titration of doses every 2–4 weeks and the association of another antihypertensive (e.g., thiazide diuretic, ACEI or ARB) if blood pressure control is not achieved (BP < 90th percentile) or in order to increase adherence, respectively to decrease the cost (45).

In pediatric practice, the following substances are approved, used in standard pediatric doses (Table 3):

**Table 3:** Antihypertensive substances approved in pediatric practice (73, 118).

Class	Substance	Remarks
ACEI	Benazepril Captopril Enalapril Fosinopril Lisinopril Quinapril	Contraindicated in pregnancy; they can affect potassium balance, kidney function and cause coughing
ARB	Losartan Ibesartan	Contraindicated in pregnancy; better tolerated; Exists in the form of suspension
CCB	Amlodipine Felodipine Isradipine Nifedipine	They have a vasodilating action, promoting diuresis
Beta blockers	Bisoprolol Metropolitan Propanolol Labetalol	Non-cardioselective agents are not administered when asthma coexists; can induce hypoglycemia and affect physical capacity
Diuretics	Hydrochlorothiazide Chlorthalidone Furosemide Spironolactone Triamterene Amiloride	Special attention must be paid to the alteration of potassium metabolism, depending on the therapeutic class
Centrally acting antiadrenergics	Clonidine	Can cause xerostomia, sedation, nasal obstruction
Peripheral blockers	Prazosin Doxazosin	Presents a risk of syncope and water retention
Vasodilators	Hydralazine Minoxidil	Can cause tachycardia, water retention, syndrome lupus like (H) or hypertrichosis (M)

ACEI, angiotensin-converting enzyme inhibitors; ARB, angiotensin receptor blockers; CCB, calcium channel blockers; H, hydralazine; M, minoxidil.

The poor response of African Americans to ACEI is known, therefore, in their case, the use of a thiazide diuretic or a calcium channel blocker is recommended in the initial therapy (45).

Similar to the other comorbidities present in the pediatric MetS, the therapeutic market targeting arterial hypertension is constantly developing, the newest products coming into focus being endothelin receptor antagonists (Darusentan) and renin inhibitors (Aliskiren) (106).

### 5.8 Management of non-alcoholic fatty liver disease recently known as metabolic fatty liver disease

Liver damage is also a constituent pathology of MetS in children. Until recently, liver damage following the excessive accumulation of fats from a non-alcoholic cause was called NAFLD. This varies from steatosis to steatohepatitis, progressing over time to fibrosis, cirrhosis or even liver failure. The diagnosis in children is mostly one of exclusion, made by excluding the history of chronic alcohol consumption, as well as viral infections, genetic diseases, medications and other metabolic disorders. Confirmation of the diagnosis is made with the help of imaging methods (ultrasound, MRI) or liver biopsy. Recently, it was proposed to change the terminology from NAFLD to MAFLD. This involves recognizing the direct link between fat accumulation in the liver and metabolic dysfunctions. Consequently, in addition to imaging and histopathological diagnostic methods, we find the need to objectify metabolic dysfunctions adapted to age (increased BMI, type 2 diabetes, insulin resistance, dyslipidemia, hypertension or metabolic syndrome) (125, 126). Due to the variation in diagnostic criteria between the two entities, Xing et al. states that the MAFLD criteria can identify more obese children than the NAFLD criteria. The authors estimate that approximately 19% of children with NAFLD do not have MAFLD, a percentage influenced by the secondary causes of steatosis, not covered by the secondary terminology (127). However, MAFLD is considered superior for reducing stigma, risk of hepatic and extrahepatic mortality, disease associations, and for screening high-risk individuals (126).

Mann et al. underlines, with the help of a meta-analysis that included 21 randomized controlled trials, the heterogeneity of the therapeutic means used in NAFLD. This aspect is partly due to the reporting of the effectiveness of the therapy with the help of liver markers and not histological aspects (the two criteria being met only in the case of antioxidants) (128). Due to the close connection with increased body weight and dyslipidemia, the main component of the treatment is the association between weight loss by approaching a healthy lifestyle (previously described) or resorting to bariatric surgery when there are criteria for this. It is mentioned here that rapid weight loss can worsen fibrosis and steatosis. The other strategies targeting the associated physiopathological mechanisms such as *Lactobacillus*-based probiotics, N-acetylcysteine, nuclear receptor agonists (Pioglitazone), GLP-1 agonists (Liraglutide), DDP-4 inhibitors (Sitagliptin), omega-3 fatty acids (docosahexaenoic acid) or bile acids (obeticholic acid) and vitamin E, with/without vitamin D in cases of deficiency remain under research (40, 44, 45, 129–132). Uncertain effects seem to appear also in the case of Metformin through the reduction of alanine amino-transferase (ALT) and the positive modulation of histological dynamics (45, 132). Antifibrotic agents must be added to these because, despite the possible clinical-biological reversal of the steatohepatitis process, patients may still present with fibrosis (129).

### 5.9 Impact of antipsychotics

In pediatric MetS therapy, possible drug interactions must also be taken into account. Data from the literature of recent years show an increase in its incidence among patients, especially children, under treatment with second-generation antipsychotics (Clozapine, Olanzapine, and Risperidone) for neuropsychiatric disorders - e.g., behavioral disorders or schizophrenia. The clinical manifestations seem to follow each other in a vicious circle, the starting point of which is represented by the imbalance of appetite as a result of the vicious integration of the regulation signals at the level of the arcuate nucleus or the disturbance of the intestinal microbiota. From a therapeutic point of view, the effectiveness of obtaining control of the symptoms with the help of the means stated above remains controversial. However, food polyphenols are recommended in the control of obesity, inflammation and insulin resistance (133–136). Early recognition of MetS, especially among children who receive antipsychotic treatment for long periods, is therefore an essential component in clinical practice. The screening targets the same lines as in the classic syndrome, namely the measurement of body weight, waist circumference, lipid profile and the value of blood glucose and insulin after digestive rest (fasting) (137).

### 5.10 Particularities and practical directions

The particular aspects of the evolutionary course in pediatric MetS, which must be considered by the clinician for the purpose of early diagnosis and optimal treatment, include its entanglement with other pathologies/medical interventions such as hematopoietic stem cell transplantation, respectively liver. In this situation, the pathology is precipitated as a consequence of the existence of risk factors such as non-alcoholic fatty liver, bone or renal complications, but also the use of immunotherapy, corticotherapy or calcineurin inhibitors (138). It seems that the risk of the occurrence of the comorbidities that make up pediatric MetS is higher starting with the early post-transplant period and spreads over 5 to 10 years after it, having an undulating, individual incidence over time (139). However, the theory remains controversial, Kosola et al. reporting, through a cross-sectional study, a prevalence of pediatric MetS components similar to that found in the general population, namely 20% for overweight, 24% for hypertension, 14% for fasting hyperglycemia, 9% for hypertriglyceridemia and 23% for lowering of HDL-cholesterol. The authors do not highlight a significant correlation with the use of immunosuppression (140). Regarding the metabolic consequences of stem cell transplantation, Paris et al. recorded a prevalence of pediatric MetS of 32%, significantly higher than the healthy population, doubled by the strong association between it and the use of corticotherapy (141).

At the same time, in agreement with the above, knowledge and practical integration of the principles of the Mediterranean diet must represent a desire of modern medicine. In this sense, Table 4 shows the most used foods, their recommended frequency, the constituent nutritional principles, as well as the biological functions performed.

**Table 4:** The Mediterranean diet - between theory and practice (142, 143).

Feature described	Recommendation		
Food	Frequent	Moderate	Rare
	Olive oil, vegetables, fruits, nuts, legumes, unprocessed grains	Fish, red wine, dairy	Poultry, red meat, processed red meat products
	Food principles: fatty acids omega 3, omega 9, fiber, calcium, magnesium, potassium, proteins, complex carbohydrates, vitamins A, C, E, quercetin, resveratrol, rosmarinic acid, oleuropein, hydroxytyrosol, tyrosol, eleocanthal, ligstroside, naringenin, apigenin, kaempferol, hesperidin, ellagic acid		
Frequency of meals	Every main meal	Every day	Weekly
	Fruits 1–2 / Vegetables ≥2 servings Olive oil Bread/ pasta/ rice/ couscous Other cereals 1–2 servings	Milk 2 servings Olives/nuts/seeds Herbs/spices/garlic/onions	Potatoes ≤3 servings White meat 2 servings Red meat <2 servings Processed meat ≤1 servings Fish / Seafood ≥2 servings Eggs 2–4 servings Vegetables ≥2 servings Sweets ≤2 servings
Means of action and effects	Vitamins (A, C, E)	Decrease inflammation and increase antioxidant capacity → improve β cell functions → reduce endothelial dysfunction	
	Mineral	Ca and Mg: Promotes vasodilation → decrease blood pressure K: reduces sodium reabsorption → decrease blood pressure	
	Polyphenols	Inhibits NFKB and ACE Activates SIRT-1 and AMPK Reduce AGEs Improve energy metabolism Exerts prebiotic effects → decrease inflammation, blood pressure and oxidative stress → increase lipolysis, insulin sensitivity and energy consumption → lowers LDL and increases HDL cholesterol, improving dyslipidemia	
	Fatty acids	Decrease inflammation Reduce ghrelin secretion Reduce platelet aggregation Decrease LDL and increase HDL Inhibits ACE → improve β cell functions and decrease endothelial dysfunction → reduce appetite → improves dyslipidemia → decrease blood pressure	
	Dietary fiber	Soluble fibers: Increase gastric emptying time Increase the absorption of macronutrients Increase SCFA synthesis Decrease the reabsorption of bile acids → increase satiety → decrease the absorption of macronutrients → improve the intestinal microbiota → lower plasma cholesterol Insoluble fibers: Increase chewing time Decrease colon transit time → increase satiety → decrease the absorption of macronutrients	
Direct benefits	Decreases visceral and abdominal obesity Decreases insulin resistance Decreases dyslipidemia It reduces blood pressure		

*NFKB: Nuclear factor kappa-light-chain-enhancer of activated B cells, ACE: Angiotensin-converting enzyme, SIRT-1: Sirtuin 1, AMPK: Adenosine monophosphate-activated protein kinase, AGE: Advanced Glycation End Products, LDL: Low-density lipoproteins, HDL: high-density lipoproteins, SCFA: Short-chain fatty acids.

### 5.11 Conclusion

In conclusion, MetS represents a touchstone for the clinician, especially at the pediatric age, partly due to the entanglement of multiple physiopathological processes that promote and potentiate each other. The optimal knowledge of preventive and therapeutic measures is therefore vital in the evolutionary course of affected children, with resonance both from a psychological and social point of view, as well as in the quality of life of the future adult. The narrative exposition fulfilled its purpose of creating a schematic description of the general characteristics related to pediatric MetS, the location of the pathology in the current epidemiological context, its diagnosis, pathogenic mechanisms underlying the development, the main risk factors encountered and the way in which they can be modulated for a beneficial purpose. The central point of interest was the display of the main directions used in the approach to the patient with pediatric MetS, starting from lifestyle changes and culminating with pharmacotherapy aimed at the various associated comorbidities - obesity, dyslipidemia, hypertension, changes in insulin metabolism or non-fatty liver -alcoholic. For this purpose, the main pharmacological products approved or in the process of being approved in pediatrics were presented, doubled by doses, mechanisms of action, clinical results, but also frequent adverse effects. The novelty brought into discussion is the approach to the fecal microbiota (probiotics, transplant) in modulating the metabolic imbalance and emphasizing the additional risk of the use of antipsychotics in inducing disturbances. Attention was also drawn to the implications of stem cell or liver transplantation in disrupting the body’s homeostasis, especially the pathology targeted in this study. Therefore, pediatric MetS can be considered a complex syndrome whose knowledge and prevention are a pillar in the optimal development of society. For this purpose, we draw attention to the need to implement standardized screening programs, to popularize the benefits of a healthy lifestyle, but also to form “attack” protocols at the level of each region with the aim of eliminating the variability of therapeutic results induced by cultural and racial differences.

## References

[1] DeBoer MD. Assessing and managing the metabolic syndrome in children and adolescents. Nutrients. (2019) 11:1788. doi: 10.3390/nu11081788, PMID: 31382417 PMC6723651

[2] Dündar İ Akıncı A. Prevalence of type 2 diabetes mellitus, metabolic syndrome, and related morbidities in overweight and obese children. J Pediatr Endocrinol Metab. (2022) 35:435–41. doi: 10.1515/jpem-2021-0271, PMID: 35026882

[3] Ishaque A. Metabolic syndrome in children: an emerging epidemic. J Pak Med Assoc. (2021) 71:396. 33819213

[4] Fazeli M Mohammad-Zadeh M Meshkat Z Ghazizadeh H Barati E Ferns GA. Metabolic syndrome in children and adolescents: looking to new markers. Curr Treat Options Peds. (2021) 7:152–66. doi: 10.1007/s40746-021-00226-7

[5] Weihe P Weihrauch-Blüher S. Metabolic syndrome in children and adolescents: diagnostic criteria, therapeutic options and perspectives. Curr Obes Rep. (2019) 8:472–9. doi: 10.1007/s13679-019-00357-x, PMID: 31691175

[6] Serbis A Giapros V Galli-Tsinopoulou A Siomou E. Metabolic syndrome in children and adolescents: is there a universally accepted definition? Does it matter? Metab Syndr Relat Disord. (2020) 18:462–70. doi: 10.1089/met.2020.0076, PMID: 32795106

[7] Tagi VM Samvelyan S Chiarelli F. Metabolic syndrome in children. Minerva Pediatr. (2020) 72:312–25. doi: 10.23736/S0026-4946.20.05834-X 32274915

[8] He M Wang J Liang Q Li M Guo H Wang Y. Time-restricted eating with or without low-carbohydrate diet reduces visceral fat and improves metabolic syndrome: a randomized trial. Cell Rep Med. (2022) 3:100777. doi: 10.1016/j.xcrm.2022.100777, PMID: 36220069 PMC9589024

[9] Friend A Craig L Turner S. The prevalence of metabolic syndrome in children: a systematic review of the literature. Metab Syndr Relat Disord. (2013) 11:71–80. doi: 10.1089/met.2012.0122 23249214

[10] Noubiap JJ Nansseu JR Lontchi-Yimagou E Nkeck JR Nyaga UF Ngouo AT. Global, regional, and country estimates of metabolic syndrome burden in children and adolescents in 2020: a systematic review and modelling analysis. Lancet Child Adolesc Health. (2022) 6:158–70. doi: 10.1016/S2352-4642(21)00374-6, PMID: 35051409

[11] Bitew ZW Alemu A Ayele EG Tenaw Z Alebel A Worku T. Metabolic syndrome among children and adolescents in low and middle income countries: a systematic review and meta-analysis. Diabetol Metab Syndr. (2020) 12:93. doi: 10.1186/s13098-020-00601-8, PMID: 33117455 PMC7590497

[12] Jia G Wu CC Su CH. Dietary inflammatory index and metabolic syndrome in US children and adolescents: evidence from NHANES 2001–2018. Nutr Metab (Lond). (2022) 19:39. doi: 10.1186/s12986-022-00673-5, PMID: 35698152 PMC9195322

[13] Codazzi V Frontino G Galimberti L Giustina A Petrelli A. Mechanisms and risk factors of metabolic syndrome in children and adolescents. Endocrine. (2024) 84:16–28. doi: 10.1007/s12020-023-03642-x, PMID: 38133765 PMC10987369

[14] Guzmán-Guzmán IP Salgado-Bernabé AB Muñoz Valle JF Vences-Velázquez A Parra-Rojas I. Prevalence of metabolic syndrome in children with and without obesity. Med Clin (Barc). (2015) 144:198–203. doi: 10.1016/j.medcli.2013.10.033, PMID: 24721677

[15] Wan Mahmud Sabri WMN Mohamed RZ Yaacob NM Hussain S. Prevalence of metabolic syndrome and its associated risk factors in pediatric obesity. J ASEAN Fed Endocr Soc. (2022) 37:24–30. doi: 10.15605/jafes.037.01.05, PMID: 35800595 PMC9242664

[16] Lepe A de Kroon MLA de Winter AF Reijneveld SA. Alternative pediatric metabolic syndrome definitions impact prevalence estimates and socioeconomic gradients. Pediatr Res. (2021) 90:694–700. doi: 10.1038/s41390-020-01331-3, PMID: 33446919

[17] Litwin M Kułaga Z. Obesity, metabolic syndrome, and primary hypertension. Pediatr Nephrol. (2021) 36:825–37. doi: 10.1007/s00467-020-04579-3, PMID: 32388582 PMC7910261

[18] Lee L Sanders RA. Metabolic syndrome. Pediatr Rev. (2012) 33:459–68. doi: 10.1542/pir.33.10.459 23027600 PMC4109314

[19] Weiss R Bremer AA Lustig RH. What is metabolic syndrome, and why are children getting it? Ann N Y Acad Sci. (2013) 1281:123–40. doi: 10.1111/nyas.12030, PMID: 23356701 PMC3715098

[20] Wu J Zhang H Yang L Shao J Chen D Cui N. Sedentary time and the risk of metabolic syndrome: a systematic review and dose-response meta-analysis. Obes Rev. (2022) 23:e13510. doi: 10.1111/obr.13510, PMID: 36261077

[21] Bizerea-Moga TO Pitulice L Pantea CL Olah O Marginean O Moga TV. Extreme birth weight and metabolic syndrome in children. Nutrients. (2022) 14:204. doi: 10.3390/nu14010204, PMID: 35011079 PMC8746946

[22] Panera N Mandato C Crudele A Bertrando S Vajro P Alisi A. Genetics, epigenetics and transgenerational transmission of obesity in children. Front Endocrinol (Lausanne). (2022) 13:1006008. doi: 10.3389/fendo.2022.1006008, PMID: 36452324 PMC9704419

[23] Darendeliler F. IUGR: genetic influences, metabolic problems, environmental associations/triggers, current and future management. Best Pract Res Clin Endocrinol Metab. (2019) 33:101260. doi: 10.1016/j.beem.2019.01.001, PMID: 30709755

[24] McCracken E Monaghan M Sreenivasan S. Pathophysiology of the metabolic syndrome. Clin Dermatol. (2018) 36:14–20. doi: 10.1016/j.clindermatol.2017.09.004 29241747

[25] Rochlani Y Pothineni NV Kovelamudi S Mehta JL. Metabolic syndrome: pathophysiology, management, and modulation by natural compounds. Ther Adv Cardiovasc Dis. (2017) 11:215–25. doi: 10.1177/1753944717711379, PMID: 28639538 PMC5933580

[26] Prasun P. Mitochondrial dysfunction in metabolic syndrome. Biochim Biophys Acta Mol basis Dis. (2020) 1866:165838. doi: 10.1016/j.bbadis.2020.165838 32428560

[27] Christian Flemming GM Bussler S Körner A Kiess W. Definition and early diagnosis of metabolic syndrome in children. J Pediatr Endocrinol Metab. (2020) 33:821–33. doi: 10.1515/jpem-2019-0552 32568734

[28] Abiri B Valizadeh M Amini S Kelishadi R Hosseinpanah F. Risk factors, cutoff points, and definition of metabolically healthy/unhealthy obesity in children and adolescents: a scoping review of the literature. Obes Rev. (2023) 24:e13548. doi: 10.1111/obr.13548, PMID: 36624970

[29] Lopez Lucas MJ Barja S Villarroel Del Pino L Arnaiz P Mardones F. Cardiometabolic risk in children with severe obesity. Nutr Hosp. (2022) 39:290–7. doi: 10.20960/nh.03829 34913346

[30] Daye M Selver Eklioglu B Atabek ME. Relationship of acanthosis nigricans with metabolic syndrome in obese children. J Pediatr Endocrinol Metab. (2020) 33:1563–8. doi: 10.1515/jpem-2020-0154 33581705

[31] Gaines J Vgontzas AN Fernandez-Mendoza J Bixler EO. Obstructive sleep apnea and the metabolic syndrome: the road to clinically-meaningful phenotyping, improved prognosis, and personalized treatment. Sleep Med Rev. (2018) 42:211–9. doi: 10.1016/j.smrv.2018.08.009, PMID: 30279095 PMC6221996

[32] Ighbariya A Weiss R. Insulin resistance, prediabetes, metabolic syndrome: what should every pediatrician know? J Clin Res Pediatr Endocrinol. (2017) 9:49–57. doi: 10.4274/jcrpe.2017.S005, PMID: 29280741 PMC5790325

[33] Steele CE Morrell D Evans M. Metabolic syndrome and inflammatory skin conditions. Curr Opin Pediatr. (2019) 31:515–22. doi: 10.1097/MOP.0000000000000790 31169544

[34] Vizzuso S Del Torto A Dilillo D Calcaterra V Di Profio E Leone A. Visceral adiposity index (VAI) in children and adolescents with obesity: no association with daily energy intake but promising tool to identify metabolic syndrome (MetS). Nutrients. (2021) 13:413. doi: 10.3390/nu13020413, PMID: 33525454 PMC7911630

[35] Guardamagna O Baracco V Abello F Bona G. Identification and management of dyslipidemic children. Minerva Pediatr. (2009) 61:391–8. PMID: 19752848

[36] Medeiros AM Alves AC Aguiar P Bourbon M. Pediatric investigators of the Portuguese familial hypercholesterolemia study. Cardiovascular risk assessment of dyslipidemic children: analysis of biomarkers to identify monogenic dyslipidemia. J Lipid Res. (2014) 55:947–55. doi: 10.1194/jlr.P043182, PMID: 24627126 PMC3995472

[37] de Simone G Mancusi C Hanssen H Genovesi S Lurbe E Parati G. Hypertension in children and adolescents. Eur Heart J. (2022) 43:3290–301. doi: 10.1093/eurheartj/ehac328 35896123

[38] Ogawa W Araki E Ishigaki Y Hirota Y Maegawa H Yamauchi T. New classification and diagnostic criteria for insulin resistance syndrome. Endocr J. (2022) 69:107–13. doi: 10.1507/endocrj.EJ21-0725 35110500

[39] Liebe R Esposito I Bock HH Vom Dahl S Stindt J Baumann U. Diagnosis and management of secondary causes of steatohepatitis. J Hepatol. (2021) 74:1455–71. doi: 10.1016/j.jhep.2021.01.045 33577920

[40] Brecelj J Orel R. Non-alcoholic fatty liver disease in children. Medicina (Kaunas). (2021) 57:719. doi: 10.3390/medicina57070719, PMID: 34357000 PMC8304730

[41] Ravaja N Keltikangas-Järvinen L. Temperament and metabolic syndrome precursors in children: a three-year follow-up. Prev Med. (1995) 24:518–27. doi: 10.1006/pmed.1995.1082, PMID: 8524728

[42] Albert Pérez E Mateu Olivares V Martínez-Espinosa RM Molina Vila MD Reig G-GM. New insights about how to make an intervention in children and adolescents with metabolic syndrome: diet, exercise vs. changes in body composition. A systematic review of RCT. Nutrients. (2018) 10:878. doi: 10.3390/nu10070878, PMID: 29986479 PMC6073719

[43] Urbina E. Noninvasive assessment of target organ injury in children with the metabolic syndrome. J Cardiometab Syndr. (2006) 1:277–81. doi: 10.1111/j.1559-4564.2006.05799.x, PMID: 17679807

[44] Fornari E Maffeis C. Treatment of metabolic syndrome in children. Front Endocrinol (Lausanne). (2019) 10:702. doi: 10.3389/fendo.2019.00702, PMID: 31681173 PMC6803446

[45] Tagi VM Samvelyan S Chiarelli F. (2020). Treatment of metabolic syndrome in children. Horm Res Paediatr. (2020) 93:215–25. doi: 10.1159/000510941 33017828

[46] Andersen CJ Fernandez ML. Dietary strategies to reduce metabolic syndrome. Rev Endocr Metab Disord. (2013) 14:241–54. doi: 10.1007/s11154-013-9251-y 23943309

[47] Papadaki A Nolen-Doerr E Mantzoros CS. The effect of the Mediterranean diet on metabolic health: a systematic review and Meta-analysis of controlled trials in adults. Nutrients. (2020) 12:3342. doi: 10.3390/nu12113342, PMID: 33143083 PMC7692768

[48] Martino F Puddu PE Lamacchia F Colantoni C Zanoni C Barillà F. Mediterranean diet and physical activity impact on metabolic syndrome among children and adolescents from southern Italy: contribution from the Calabrian sierras community study (CSCS). Int J Cardiol. (2016) 225:284–8. doi: 10.1016/j.ijcard.2016.10.008 27744204

[49] Seral-Cortes M Larruy-García A De Miguel-Etayo P Labayen I Moreno LA. Mediterranean diet and genetic determinants of obesity and metabolic syndrome in European children and adolescents. Genes (Basel). (2022) 13:420. doi: 10.3390/genes13030420, PMID: 35327974 PMC8954235

[50] Asoudeh F Fallah M Aminianfar A Djafarian K Shirzad N Clark CCT. The effect of Mediterranean diet on inflammatory biomarkers and components of metabolic syndrome in adolescent girls. J Endocrinol Investig. (2023) 46:1995–2004. doi: 10.1007/s40618-023-02027-1, PMID: 36795242

[51] Masini A Dallolio L Sanmarchi F Lovecchio F Falato M Longobucco Y. Adherence to the Mediterranean diet in children and adolescents and association with multiple outcomes: an umbrella review. Healthcare. (2024) 12:449. doi: 10.3390/healthcare12040449, PMID: 38391824 PMC10887852

[52] López-Gil JF García-Hermoso A Martínez-González MÁ Rodríguez-Artalejo F. Mediterranean diet and Cardiometabolic biomarkers in children and adolescents: a systematic review and Meta-analysis. JAMA Netw Open. (2024) 7:e2421976. doi: 10.1001/jamanetworkopen.2024.21976, PMID: 38995643 PMC11245727

[53] George ES Gavrili S Itsiopoulos C Manios Y Moschonis G. Poor adherence to the Mediterranean diet is associated with increased likelihood of metabolic syndrome components in children: the healthy growth study. Public Health Nutr. (2021) 24:2823–33. doi: 10.1017/S1368980021001701, PMID: 33866986 PMC9884535

[54] Serra-Majem L Ribas L Ngo J Ortega RM García A Pérez-Rodrigo C. Food, youth and the Mediterranean diet in Spain. Development of KIDMED, Mediterranean diet quality index in children and adolescents. Public Health Nutr. (2004) 7:931–5. doi: 10.1079/phn2004556, PMID: 15482620

[55] Ventura EE Davis JN Alexander KE Shaibi GQ Lee W Byrd-Williams CE. Dietary intake and the metabolic syndrome in overweight Latino children. J Am Diet Assoc. (2008) 108:1355–9. doi: 10.1016/j.jada.2008.05.006, PMID: 18656576 PMC2882193

[56] Halpern A Mancini MC Magalhães ME Fisberg M Radominski R Bertolami MC. Metabolic syndrome, dyslipidemia, hypertension and type 2 diabetes in youth: from diagnosis to treatment. Diabetol Metab Syndr. (2010) 2:55. doi: 10.1186/1758-5996-2-55, PMID: 20718958 PMC2939537

[57] Gregory JW. Prevention of obesity and metabolic syndrome in children. Front Endocrinol (Lausanne). (2019) 10:669. doi: 10.3389/fendo.2019.00669, PMID: 31632348 PMC6779866

[58] Coppen AM Risser JA Vash PD. Metabolic syndrome resolution in children and adolescents after 10 weeks of weight loss. J Cardiometab Syndr. (2008) 3:205–10. doi: 10.1111/j.1559-4572.2008.00016.x, PMID: 19040588

[59] Velázquez-López L Santiago-Díaz G Nava-Hernández J Muñoz-Torres AV Medina-Bravo P Torres-Tamayo M. Mediterranean-style diet reduces metabolic syndrome components in obese children and adolescents with obesity. BMC Pediatr. (2014) 14:175. doi: 10.1186/1471-2431-14-175, PMID: 24997634 PMC4102089

[60] Pedrosa C Oliveira BM Albuquerque I Simões-Pereira C Vaz-de-Almeida MD Correia F. Markers of metabolic syndrome in obese children before and after 1-year lifestyle intervention program. Eur J Nutr. (2011) 50:391–400. doi: 10.1007/s00394-010-0148-1, PMID: 21107585

[61] Zimmermann MB Aeberli I. Dietary determinants of subclinical inflammation, dyslipidemia and components of the metabolic syndrome in overweight children: a review. Int J Obes. (2008) 32:S11–8. doi: 10.1038/ijo.2008.202, PMID: 19079275

[62] Villatoro-Santos CR Ramirez-Zea M Villamor E. Nine Mesoamerican countries metabolic syndrome (NiMeCoMeS) study group. B-vitamins and metabolic syndrome in Mesoamerican children and their adult parents. Public Health Nutr. (2021) 24:4537–45. doi: 10.1017/S1368980020003936, PMID: 33023697 PMC8024411

[63] Piuri G Zocchi M Della Porta M Ficara V Manoni M Zuccotti GV. Magnesium in obesity, metabolic syndrome, and type 2 diabetes. Nutrients. (2021) 13:320. doi: 10.3390/nu13020320, PMID: 33499378 PMC7912442

[64] Castro-Barquero S Ruiz-León AM Sierra-Pérez M Estruch R Casas R. Dietary strategies for metabolic syndrome: a comprehensive review. Nutrients. (2020) 12:2983. doi: 10.3390/nu12102983, PMID: 33003472 PMC7600579

[65] Bueno NB de Melo IS de Oliveira SL da Rocha AT. Very-low-carbohydrate ketogenic diet v. low-fat diet for long-term weight loss: a meta-analysis of randomised controlled trials. Br J Nutr. (2013) 110:1178–87. doi: 10.1017/S0007114513000548 23651522

[66] Chawla S Tessarolo Silva F Amaral Medeiros S Mekary RA Radenkovic D. The effect of low-fat and low-carbohydrate Diets on weight loss and lipid levels: a systematic review and Meta-analysis. Nutrients. (2020) 12:3774. doi: 10.3390/nu12123774, PMID: 33317019 PMC7763365

[67] Churuangsuk C Lean MEJ Combet E. Low and reduced carbohydrate diets: challenges and opportunities for type 2 diabetes management and prevention. Proc Nutr Soc. (2020) 79:1–16. doi: 10.1017/S0029665120000105, PMID: 32131904

[68] Liu AG Ford NA Hu FB Zelman KM Mozaffarian D Kris-Etherton PM. A healthy approach to dietary fats: understanding the science and taking action to reduce consumer confusion. Nutr J. (2017) 16:53. doi: 10.1186/s12937-017-0271-4, PMID: 28854932 PMC5577766

[69] Castro-Quezada I Román-Viñas B Serra-Majem L. The Mediterranean diet and nutritional adequacy: a review. Nutrients. (2014) 6:231–48. doi: 10.3390/nu6010231, PMID: 24394536 PMC3916858

[70] Barnes TL Crandell JL Bell RA Mayer-Davis EJ Dabelea D Liese AD. Change in DASH diet score and cardiovascular risk factors in youth with type 1 and type 2 diabetes mellitus: the SEARCH for diabetes in youth study. Nutr Diabetes. (2013) 3:e91. doi: 10.1038/nutd.2013.32, PMID: 24126768 PMC3817346

[71] Eisenmann JC. Aerobic fitness, fatness and the metabolic syndrome in children and adolescents. Acta Paediatr. (2007) 96:1723–9. doi: 10.1111/j.1651-2227.2007.00534.x, PMID: 17971189

[72] Misigoj-Duraković M Duraković Z. The early prevention of metabolic syndrome by physical exercise. Coll Antropol. (2009) 33:759–64. PMID: 19860101

[73] Brambilla P Pozzobon G Pietrobelli A. Physical activity as the main therapeutic tool for metabolic syndrome in childhood. Int J Obes. (2011) 35:16–28. doi: 10.1038/ijo.2010.255 21139560

[74] Guinhouya BC. Physical activity in preventing metabolic syndrome in children. Med Sci (Paris). (2009) 25:827–33. doi: 10.1051/medsci/20092510827, PMID: 19849985

[75] Ben Ounis O Elloumi M Makni E Zouhal H Amri M Tabka Z. Exercise improves the ApoB/ApoA-I ratio, a marker of the metabolic syndrome in obese children. Acta Paediatr. (2010) 99:1679–85. doi: 10.1111/j.1651-2227.2010.01920.x, PMID: 20594189

[76] Leister KR Cilhoroz BT Rosenberg J Brown EC Kim JY. Metabolic syndrome: operational definitions and aerobic and resistance training benefits on physical and metabolic health in children and adolescents. Diabetes Metab Syndr. (2022) 16:102530. doi: 10.1016/j.dsx.2022.102530, PMID: 35709585

[77] MacKenzie-Shalders K Kelly JT So D Coffey VG Byrne NM. The effect of exercise interventions on resting metabolic rate: a systematic review and meta-analysis. J Sports Sci. (2020) 38:1635–49. doi: 10.1080/02640414.2020.1754716 32397898

[78] Chomiuk T Niezgoda N Mamcarz A Śliż D. Physical activity in metabolic syndrome. Front Physiol. (2024) 15:1365761. doi: 10.3389/fphys.2024.1365761, PMID: 38440349 PMC10910017

[79] Quiroga R Nistal E Estébanez B Porras D Juárez-Fernández M Martínez-Flórez S. Exercise training modulates the gut microbiota profile and impairs inflammatory signaling pathways in obese children. Exp Mol Med. (2020) 52:1048–61. doi: 10.1038/s12276-020-0459-0, PMID: 32624568 PMC8080668

[80] Pantazi AC Balasa AL Mihai CM Chisnoiu T Lupu VV Kassim MAK. Development of gut microbiota in the first 1000 days after birth and potential interventions. Nutrients. (2023) 15:3647. doi: 10.3390/nu15163647, PMID: 37630837 PMC10457741

[81] Lupu VV Jechel E Mihai CM Mitrofan EC Lupu A Starcea IM. Connection between celiac disease and systemic lupus erythematosus in children-a development model of autoimmune diseases starting from what we inherit to what we eat. Nutrients. (2023) 15:2535. doi: 10.3390/nu15112535, PMID: 37299501 PMC10255122

[82] Lupu VV Sasaran MO Jechel E Starcea IM Ioniuc I Mocanu A. Celiac disease - a pluripathological model in pediatric practice. Front Immunol. (2024) 15:1390755. doi: 10.3389/fimmu.2024.1390755, PMID: 38715620 PMC11074362

[83] Lupu VV Butnariu LI Fotea S Morariu ID Badescu MC Starcea IM. The disease with a thousand faces and the human microbiome—a physiopathogenic intercorrelation in pediatric practice. Nutrients. (2023) 15:3359. doi: 10.3390/nu15153359, PMID: 37571295 PMC10420997

[84] Lupu VV Lupu A Jechel E Starcea IM Stoleriu G Ioniuc I. The role of vitamin D in pediatric systemic lupus erythematosus - a double pawn in the immune and microbial balance. Front Immunol. (2024) 15:1373904. doi: 10.3389/fimmu.2024.1373904, PMID: 38715605 PMC11074404

[85] Lupu A Jechel E Mihai CM Mitrofan EC Fotea S Starcea IM. The footprint of microbiome in pediatric asthma—a complex puzzle for a balanced development. Nutrients. (2023) 15:3278. doi: 10.3390/nu15143278, PMID: 37513696 PMC10384859

[86] Lupu VV Ghiciuc CM Stefanescu G Mihai CM Popp A Sasaran MO. Emerging role of the gut microbiome in post-infectious irritable bowel syndrome: a literature review. World J Gastroenterol. (2023) 29:3241–56. doi: 10.3748/wjg.v29.i21.3241, PMID: 37377581 PMC10292139

[87] Lupu VV Bratu RM Trandafir LM Bozomitu L Paduraru G Gimiga N. Exploring the microbial landscape: gut Dysbiosis and therapeutic strategies in pancreatitis—a narrative review. Biomedicines. (2024) 12:645. doi: 10.3390/biomedicines12030645, PMID: 38540258 PMC10967871

[88] Pantazi AC Kassim MAK Nori W Tuta LA Mihai CM Chisnoiu T. Clinical perspectives of gut microbiota in patients with chronic kidney disease and end-stage kidney disease: where do we stand? Biomedicines. (2023) 11:2480. doi: 10.3390/biomedicines11092480, PMID: 37760920 PMC10525496

[89] Mocanu A Bogos RA Lazaruc TI Trandafir LM Lupu VV Ioniuc I. Exploring a complex interplay: kidney-gut Axis in pediatric chronic kidney disease. Nutrients. (2023) 15:3609. doi: 10.3390/nu15163609, PMID: 37630799 PMC10457891

[90] Carrizales-Sánchez AK García-Cayuela T Hernández-Brenes C Senés-Guerrero C. Gut microbiota associations with metabolic syndrome and relevance of its study in pediatric subjects. Gut Microbes. (2021) 13:1960135. doi: 10.1080/19490976.2021.1960135, PMID: 34491882 PMC8425709

[91] Pan B Liu X Shi J Chen Y Xu Z Shi D. A Meta-analysis of microbial therapy against metabolic syndrome: evidence from randomized controlled trials. Front Nutr. (2021) 8:775216. doi: 10.3389/fnut.2021.775216, PMID: 34977119 PMC8714845

[92] Plovier H Cani PD. Microbial impact on host metabolism: opportunities for novel treatments of nutritional disorders? Microbiol Spectr. (2017) 5:2016. doi: 10.1128/microbiolspec.BAD-0002-2016, PMID: 28597812 PMC11687490

[93] Gebrayel P Nicco C Al Khodor S Bilinski J Caselli E Comelli EM. Microbiota medicine: towards clinical revolution. J Transl Med. (2022) 20:111. doi: 10.1186/s12967-022-03296-9, PMID: 35255932 PMC8900094

[94] Loy MH Usseglio J Lasalandra D Gold MA. Probiotic use in children and adolescents with overweight or obesity: a scoping review. Child Obes. (2023) 19:145–59. doi: 10.1089/chi.2022.0059 35723657

[95] Koutnikova H Genser B Monteiro-Sepulveda M Faurie JM Rizkalla S Schrezenmeir J. Impact of bacterial probiotics on obesity, diabetes and non-alcoholic fatty liver disease related variables: a systematic review and meta-analysis of randomised controlled trials. BMJ Open. (2019) 9:e017995. doi: 10.1136/bmjopen-2017-017995, PMID: 30928918 PMC6475231

[96] Green M Arora K Prakash S. Microbial medicine: prebiotic and probiotic functional foods to target obesity and metabolic syndrome. Int J Mol Sci. (2020) 21:2890. doi: 10.3390/ijms21082890, PMID: 32326175 PMC7215979

[97] Ferrarese R Ceresola ER Preti A Canducci F. Probiotics, prebiotics and synbiotics for weight loss and metabolic syndrome in the microbiome era. Eur Rev Med Pharmacol Sci. (2018) 22:7588–605. doi: 10.26355/eurrev_201811_16301, PMID: 30468509

[98] Chen JJ Wang R Li XF Wang RL. *Bifidobacterium longum* supplementation improved high-fat-fed-induced metabolic syndrome and promoted intestinal Reg I gene expression. Exp Biol Med (Maywood). (2011) 236:823–31. doi: 10.1258/ebm.2011.010399 21685239

[99] Chen J Wang R Li XF Wang RL. *Bifidobacterium adolescentis* supplementation ameliorates visceral fat accumulation and insulin sensitivity in an experimental model of the metabolic syndrome. Br J Nutr. (2012) 107:1429–34. doi: 10.1017/S0007114511004491, PMID: 21914236

[100] Zhang Z Mocanu V Cai C Dang J Slater L Deehan EC. Impact of fecal microbiota transplantation on obesity and metabolic syndrome-a systematic review. Nutrients. (2019) 11:2291. doi: 10.3390/nu11102291, PMID: 31557953 PMC6835402

[101] Chen CC Chiu CH. Current and future applications of fecal microbiota transplantation for children. Biom J. (2022) 45:11–8. doi: 10.1016/j.bj.2021.11.004, PMID: 34781002 PMC9133305

[102] Hirschler V Oestreicher K Maccallini G Aranda C. Relationship between obesity and metabolic syndrome among Argentinean elementary school children. Clin Biochem. (2010) 43:435–41. doi: 10.1016/j.clinbiochem.2009.11.003, PMID: 19913002

[103] Hirschler V Bugna J Roque M Gilligan T Gonzalez C. Does low birth weight predict obesity/overweight and metabolic syndrome in elementary school children? Arch Med Res. (2008) 39:796–802. doi: 10.1016/j.arcmed.2008.08.003, PMID: 18996294

[104] Sen Y Kandemir N Alikasifoglu A Gonc N Ozon A. Prevalence and risk factors of metabolic syndrome in obese children and adolescents: the role of the severity of obesity. Eur J Pediatr. (2008) 167:1183–9. doi: 10.1007/s00431-007-0658-x, PMID: 18205011

[105] Lupu A Fotea S Jechel E Starcea IM Ioniuc I Knieling A. Is oxidative stress - antioxidants imbalance the physiopathogenic core in pediatric obesity? Front Immunol. (2024) 15:1394869. doi: 10.3389/fimmu.2024.1394869, PMID: 39176098 PMC11338799

[106] Mallare JT Karabell AH Velasquez-Mieyer P Stender SRS Christensen ML. Current and future treatment of metabolic syndrome and type 2 diabetes in children and adolescents. Diabetes Spectrum. (2005) 18:220–8. doi: 10.2337/diaspect.18.4.220

[107] Luong DQ Oster R Ashraf AP. Metformin treatment improves weight and dyslipidemia in children with metabolic syndrome. J Pediatr Endocrinol Metab. (2015) 28:649–55. doi: 10.1515/jpem-2014-0201, PMID: 25210757

[108] Decsi T Molnár D. Insulin resistance syndrome in children: pathophysiology and potential management strategies. Paediatr Drugs. (2003) 5:291–9. doi: 10.2165/00128072-200305050-00002 12716216

[109] Rosenberg B Moran A Sinaiko AR. Insulin resistance (metabolic) syndrome in children. Panminerva Med. (2005) 47:229–44. 16489322

[110] Fotea S Ghiciuc CM Stefanescu G Cianga AL Mihai CM Lupu A. Pediatric COVID-19 and diabetes: an investigation into the intersection of two pandemics. Diagnostics (Basel). (2023) 13:2436. doi: 10.3390/diagnostics13142436, PMID: 37510181 PMC10378192

[111] Nelson RA Bremer AA. Insulin resistance and metabolic syndrome in the pediatric population. Metab Syndr Relat Disord. (2010) 8:1–14. doi: 10.1089/met.2009.0068 19943799

[112] Gidding SS. Dyslipidemia in the metabolic syndrome in children. J Cardiometab Syndr. (2006) 1:282–5. doi: 10.1111/j.1559-4564.2006.05798.x 17679808

[113] Holmes KW Kwiterovich PO Jr. Treatment of dyslipidemia in children and adolescents. Curr Cardiol Rep. (2005) 7:445–56. doi: 10.1007/s11886-005-0063-x 16256015

[114] Kennedy MJ Jellerson KD Snow MZ Zacchetti ML. Challenges in the pharmacologic management of obesity and secondary dyslipidemia in children and adolescents. Paediatr Drugs. (2013) 15:335–42. doi: 10.1007/s40272-013-0028-2, PMID: 23677836 PMC3781297

[115] Sarti C Gallagher J. The metabolic syndrome: prevalence, CHD risk, and treatment. J Diabetes Complicat. (2006) 20:121–32. doi: 10.1016/j.jdiacomp.2005.06.014 16504841

[116] Khoury M Urbina EM. Hypertension in adolescents: diagnosis, treatment, and implications. Lancet Child Adolesc Health. (2021) 5:357–66. doi: 10.1016/S2352-4642(20)30344-8 33711291

[117] Flynn JT Kaelber DC Baker-Smith CM Blowey D Carroll AE Daniels SR. Clinical practice guideline for screening and management of high blood pressure in children and adolescents. Pediatrics. (2018) 142:e20181739. doi: 10.1542/peds.2018-1739 28827377

[118] Puri M Flynn JT. Management of hypertension in children and adolescents with the metabolic syndrome. J Cardiometab Syndr. (2006) 1:259–68. doi: 10.1111/j.1559-4564.2006.05801.x 17679805

[119] Guzman-Limon M Samuels J. Pediatric hypertension: diagnosis, evaluation, and treatment. Pediatr Clin N Am. (2019) 66:45–57. doi: 10.1016/j.pcl.2018.09.001 30454750

[120] Tran AH Urbina EM. Hypertension in children. Curr Opin Cardiol. (2020) 35:376–80. doi: 10.1097/HCO.0000000000000744 32371621

[121] Litwin M Michałkiewicz J Gackowska L. Primary hypertension in children and adolescents is an immuno-metabolic disease with hemodynamic consequences. Curr Hypertens Rep. (2013) 15:331–9. doi: 10.1007/s11906-013-0360-5, PMID: 23737217 PMC3712132

[122] Lancarotte I Nobre MR. Primordial and primary prevention programs for cardiovascular diseases: from risk assessment through risk communication to risk reduction. A review of the literature. Clinics (Sao Paulo). (2016) 71:667–78. doi: 10.6061/clinics/2016(11)09, PMID: 27982169 PMC5108165

[123] Asghari G Yuzbashian E Mirmiran P Hooshmand F Najafi R Azizi F. Dietary approaches to stop hypertension (DASH) dietary pattern is associated with reduced incidence of metabolic syndrome in children and adolescents. J Pediatr. (2016) 174:178–184.e1. doi: 10.1016/j.jpeds.2016.03.077, PMID: 27156186

[124] Lari A Sohouli MH Fatahi S Cerqueira HS Santos HO Pourrajab B. The effects of the dietary approaches to stop hypertension (DASH) diet on metabolic risk factors in patients with chronic disease: a systematic review and meta-analysis of randomized controlled trials. Nutr Metab Cardiovasc Dis. (2021) 31:2766–78. doi: 10.1016/j.numecd.2021.05.030, PMID: 34353704

[125] Xian YX Weng JP Xu F Mafld VS. NAFLD: shared features and potential changes in epidemiology, pathophysiology, diagnosis, and pharmacotherapy. Chin Med J. (2020) 134:8–19. doi: 10.1097/CM9.0000000000001263, PMID: 33323806 PMC7862804

[126] Gofton C Upendran Y Zheng MH George J. MAFLD: How is it different from NAFLD? Clin Mol Hepatol. (2023) 29:S17–31. doi: 10.3350/cmh.2022.0367, PMID: 36443926 PMC10029949

[127] Xing Y Fan J Wang HJ Wang H. Comparison of MAFLD and NAFLD characteristics in children. Children (Basel). (2023) 10:560. doi: 10.3390/children10030560, PMID: 36980118 PMC10047180

[128] Mann JP Tang GY Nobili V Armstrong MJ. Evaluations of lifestyle, dietary, and pharmacologic treatments for pediatric nonalcoholic fatty liver disease: a systematic review. Clin Gastroenterol Hepatol. (2019) 17:1457–1476.e7. doi: 10.1016/j.cgh.2018.05.023, PMID: 29857146

[129] Neuschwander-Tetri BA. Non-alcoholic fatty liver disease. BMC Med. (2017) 15:45. doi: 10.1186/s12916-017-0806-8, PMID: 28241825 PMC5330146

[130] Widhalm K Ghods E. Nonalcoholic fatty liver disease: a challenge for pediatricians. Int J Obes. (2010) 34:1451–67. doi: 10.1038/ijo.2010.185, PMID: 20838401

[131] Pacifico L Nobili V Anania C Verdecchia P Chiesa C. Pediatric nonalcoholic fatty liver disease, metabolic syndrome and cardiovascular risk. World J Gastroenterol. (2011) 17:3082–91. doi: 10.3748/wjg.v17.i26.3082, PMID: 21912450 PMC3158407

[132] Sundaram SS Zeitler P Nadeau K. The metabolic syndrome and nonalcoholic fatty liver disease in children. Curr Opin Pediatr. (2009) 21:529–35. doi: 10.1097/MOP.0b013e32832cb16f, PMID: 19444112 PMC3073840

[133] Rojo LE Gaspar PA Silva H Risco L Arena P Cubillos-Robles K. Metabolic syndrome and obesity among users of second generation antipsychotics: a global challenge for modern psychopharmacology. Pharmacol Res. (2015) 101:74–85. doi: 10.1016/j.phrs.2015.07.022, PMID: 26218604

[134] Cernea S Dima L Correll CU Manu P. Pharmacological Management of Glucose Dysregulation in patients treated with second-generation antipsychotics. Drugs. (2020) 80:1763–81. doi: 10.1007/s40265-020-01393-x 32930957

[135] Dori N Green T. The metabolic syndrome and antipsychotics in children and adolescents. Harefuah. (2011) 150:791. PMID: 22111125

[136] Libowitz MR Nurmi EL. The burden of antipsychotic-induced weight gain and metabolic syndrome in children. Front Psych. (2021) 12:623681. doi: 10.3389/fpsyt.2021.623681, PMID: 33776816 PMC7994286

[137] Lydon A Vallely J Tummon A Maher S Sabri S McLoughlin J. Routine screening and rates of metabolic syndrome in patients treated with clozapine and long-acting injectable antipsychotic medications: a cross-sectional study. Ir J Psychol Med. (2021) 38:40–8. doi: 10.1017/ipm.2020.12, PMID: 32204737

[138] Nobili V de Ville de Goyet J. Pediatric post-transplant metabolic syndrome: new clouds on the horizon. Pediatr Transplant. (2013) 17:216–23. doi: 10.1111/petr.12065 23496113

[139] Rothbaum Perito E Lau A Rhee S Roberts JP Rosenthal P. Posttransplant metabolic syndrome in children and adolescents after liver transplantation: a systematic review. Liver Transpl. (2012) 18:1009–28. doi: 10.1002/lt.23478, PMID: 22641460 PMC3429630

[140] Kosola S Lampela H Makisalo H Lohi J Arola J Jalanko H. Metabolic syndrome after pediatric liver transplantation. Liver Transpl. (2014) 20:1185–92. doi: 10.1002/lt.23931 24923737

[141] Paris C Yates L Lama P Zepeda AJ Gutiérrez D Palma J. Evaluation of metabolic syndrome after hematopoietic stem cell transplantation in children and adolescents. Pediatr Blood Cancer. (2012) 59:306–10. doi: 10.1002/pbc.24104, PMID: 22302361

[142] Dayi T Ozgoren M. Effects of the Mediterranean diet on the components of metabolic syndrome. J Prev Med Hyg. (2022) 63:E56–64. doi: 10.15167/2421-4248/jpmh2022.63.2S3.2747, PMID: 36479500 PMC9710414

[143] Santos-Buelga C González-Manzano S González-Paramás AM. Wine, Polyphenols, and Mediterranean Diets. What Else is there to say? Molecules. (2021) 26:5537. doi: 10.3390/molecules26185537, PMID: 34577008 PMC8468969

